# Correction: Testing strategic pluralism: The roles of attractiveness and competitive abilities to understand conditionality in men’s short-term reproductive strategies

**DOI:** 10.1371/journal.pone.0253362

**Published:** 2021-06-11

**Authors:** Oriana Figueroa, Jose Antonio Muñoz-Reyes, Carlos Rodriguez-Sickert, Nohelia Valenzuela, Paula Pavez, Oriana Ramírez-Herrera, Miguel Pita, David Diaz, Ana Belén Fernández-Martínez, Pablo Polo

There is an error in affiliation 1 for author Oriana Figueroa. The correct affiliation 1 is: Doctorado en Ciencias de la Complejidad Social, Centro de Investigación en Complejidad Social, Facultad de Gobierno, Universidad del Desarrollo, Santiago, Chile.

There is an error in affiliation 6 for author David Diaz. The correct affiliation 1 is: Centro de Investigación en Complejidad Social, Facultad de Gobierno, Universidad de Desarrollo, Santiago, Chile.
