# Correction: Correction: Testing strategic pluralism: The roles of attractiveness and competitive abilities to understand conditionality in men’s short-term reproductive strategies

**DOI:** 10.1371/journal.pone.0256192

**Published:** 2021-08-10

**Authors:** The PLOS ONE Staff

There is an error in affiliation 6 for author David Diaz in the correction published on June 11, 2021. The correct affiliation 6 is: Facultad de Economía y Negocios, Universidad de Chile, Santiago, Chile. The publisher apologizes for the error.

## References

[1] Figueroa O, Muñoz-Reyes JA, Rodriguez-Sickert C, Valenzuela N, Pavez P, Ramírez-Herrera O, et al. (2020) Testing strategic pluralism: The roles of attractiveness and competitive abilities to understand conditionality in men’s short-term reproductive strategies. PLoS ONE 15(8): e0237315. 10.1371/journal.pone.0237315 32866153 PMC7458284

[2] Figueroa O, Muñoz-Reyes JA, Rodriguez-Sickert C, Valenzuela N, Pavez P, Ramírez-Herrera O, et al. (2021) Correction: Testing strategic pluralism: The roles of attractiveness and competitive abilities to understand conditionality in men’s short-term reproductive strategies. PLoS ONE 16(6): e0253362. 10.1371/journal.pone.0253362 34115817 PMC8195375

