# Supplementary material for: Testing strategic pluralism: The roles of attractiveness and competitive abilities to understand conditionality in men’s short-term reproductive strategies
Source: PLoS One. 2020 Aug 31;15(8):e0237315. doi: 10.1371/journal.pone.0237315 (PMC7458284; doi:10.1371/journal.pone.0237315)
Supplement: S1 File — (PDF) [file pone.0237315.s002.pdf]

## X AXIS Descriptives

| | | | Statistic | Std. Error |
|---|---|---|---|---|
| 11,12 | Mean | | ,0002313175 | ,0000582328 |
| | 95% Confidence Interval for Mean | Lower Bound | ,0001166144 | |
| | | Upper Bound | ,0003460205 | |
| | 5% Trimmed Mean | | ,0002086020 | |
| | Median | | ,0002007919 | |
| | Variance | | ,000 | |
| | Std. Deviation | | ,0009114869 | |
| | Minimum | | -,001998748 | |
| | Maximum | | ,0030890283 | |
| | Range | | ,0050877763 | |
| | Interquartile Range | | ,0012657572 | |
| | Skewness | | ,349 | ,156 |
| | Kurtosis | | ,018 | ,310 |
| 13,14 | Mean | | ,0001915536 | ,0000536855 |
| | 95% Confidence Interval for Mean | Lower Bound | ,0000858075 | |
| | | Upper Bound | ,0002972997 | |
| | 5% Trimmed Mean | | ,0001730037 | |
| | Median | | ,0001438982 | |
| | Variance | | ,000 | |
| | Std. Deviation | | ,0008403103 | |
| | Minimum | | -,002019089 | |
| | Maximum | | ,0026421448 | |
| | Range | | ,0046612338 | |
| | Interquartile Range | | ,0011691084 | |
| | Skewness | | ,318 | ,156 |
| | Kurtosis | | -,077 | ,310 |
| 15,16 | Mean | | ,0001462272 | ,0000500013 |
| | 95% Confidence Interval for Mean | Lower Bound | ,0000477379 | |
| | | Upper Bound | ,0002447164 | |
| | 5% Trimmed Mean | | ,0001317494 | |
| | Median | | ,0000451162 | |
| | Variance | | ,000 | |
| | Std. Deviation | | ,0007826442 | |
| | Minimum | | -,002014102 | |
| | Maximum | | ,0024831282 | |
| | Range | | ,0044972299 | |
| | Interquartile Range | | ,0010149443 | |
| | Skewness | | ,294 | ,156 |

## X AXIS Descriptives

| | | | Statistic | Std. Error |
|---|---|---|---|---|
| | Kurtosis | | ,100 | ,310 |
| 17,18 | Mean | | ,0001248311 | ,0000481841 |
| | 95% Confidence Interval for Mean | Lower Bound | ,0000299213 | |
| | | Upper Bound | ,0002197409 | |
| | 5% Trimmed Mean | | ,0001123266 | |
| | Median | | ,0000579852 | |
| | Variance | | ,000 | |
| | Std. Deviation | | ,0007542000 | |
| | Minimum | | -,001791725 | |
| | Maximum | | ,0025014192 | |
| | Range | | ,0042931438 | |
| | Interquartile Range | | ,0010380833 | |
| | Skewness | | ,292 | ,156 |
| | Kurtosis | | ,291 | ,310 |
| 19,20 | Mean | | ,0001310097 | ,0000476674 |
| | 95% Confidence Interval for Mean | Lower Bound | ,0000371176 | |
| | | Upper Bound | ,0002249017 | |
| | 5% Trimmed Mean | | ,0001249323 | |
| | Median | | ,0000865113 | |
| | Variance | | ,000 | |
| | Std. Deviation | | ,0007461124 | |
| | Minimum | | -,001892891 | |
| | Maximum | | ,0023488205 | |
| | Range | | ,0042417115 | |
| | Interquartile Range | | ,0010573286 | |
| | Skewness | | ,174 | ,156 |
| | Kurtosis | | -,062 | ,310 |
| 21,22 | Mean | | ,0001433855 | ,0000504593 |
| | 95% Confidence Interval for Mean | Lower Bound | ,0000439941 | |
| | | Upper Bound | ,0002427770 | |
| | 5% Trimmed Mean | | ,0001382899 | |
| | Median | | ,0001159466 | |
| | Variance | | ,000 | |
| | Std. Deviation | | ,0007898135 | |
| | Minimum | | -,001945942 | |
| | Maximum | | ,0024383784 | |
| | Range | | ,0043843203 | |
| | Interquartile Range | | ,0011035405 | |

## X AXIS Descriptives

| | | | Statistic | Std. Error |
|---|---|---|---|---|
| | Skewness | | ,129 | ,156 |
| | Kurtosis | | ,032 | ,310 |
| 23,24 | Mean | | ,0002096760 | ,0000536118 |
| | 95% Confidence Interval for Mean | Lower Bound | ,0001040750 | |
| | | Upper Bound | ,0003152771 | |
| | 5% Trimmed Mean | | ,0002085495 | |
| | Median | | ,0001674354 | |
| | Variance | | ,000 | |
| | Std. Deviation | | ,0008391579 | |
| | Minimum | | -,002242157 | |
| | Maximum | | ,0025418873 | |
| | Range | | ,0047840446 | |
| | Interquartile Range | | ,0011129330 | |
| | Skewness | | ,032 | ,156 |
| | Kurtosis | | ,132 | ,310 |
| 25,26 | Mean | | ,0002945792 | ,0000592178 |
| | 95% Confidence Interval for Mean | Lower Bound | ,0001779358 | |
| | | Upper Bound | ,0004112225 | |
| | 5% Trimmed Mean | | ,0002867914 | |
| | Median | | ,0002200923 | |
| | Variance | | ,000 | |
| | Std. Deviation | | ,0009269056 | |
| | Minimum | | -,002786896 | |
| | Maximum | | ,0028084091 | |
| | Range | | ,0055953049 | |
| | Interquartile Range | | ,0012749785 | |
| | Skewness | | ,037 | ,156 |
| | Kurtosis | | ,313 | ,310 |
| 27,28 | Mean | | ,0003601511 | ,0000618856 |
| | 95% Confidence Interval for Mean | Lower Bound | ,0002382528 | |
| | | Upper Bound | ,0004820493 | |
| | 5% Trimmed Mean | | ,0003554005 | |
| | Median | | ,0003345247 | |
| | Variance | | ,000 | |
| | Std. Deviation | | ,0009686636 | |
| | Minimum | | -,002892938 | |
| | Maximum | | ,0032073878 | |
| | Range | | ,0061003258 | |

## X AXIS Descriptives

| | | | Statistic | Std. Error |
|---|---|---|---|---|
| | Interquartile Range | | ,0014482753 | |
| | Skewness | | -,025 | ,156 |
| | Kurtosis | | ,228 | ,310 |
| 29,30 | Mean | | ,0004260654 | ,0000626813 |
| | 95% Confidence Interval for Mean | Lower Bound | ,0003025998 | |
| | | Upper Bound | ,0005495309 | |
| | 5% Trimmed Mean | | ,0004201453 | |
| | Median | | ,0004115212 | |
| | Variance | | ,000 | |
| | Std. Deviation | | ,0009811179 | |
| | Minimum | | -,003052044 | |
| | Maximum | | ,0031976057 | |
| | Range | | ,0062496501 | |
| | Interquartile Range | | ,0014207869 | |
| | Skewness | | -,029 | ,156 |
| | Kurtosis | | ,427 | ,310 |
| 31,32 | Mean | | ,0005016958 | ,0000629827 |
| | 95% Confidence Interval for Mean | Lower Bound | ,0003776365 | |
| | | Upper Bound | ,0006257550 | |
| | 5% Trimmed Mean | | ,0004959944 | |
| | Median | | ,0005272585 | |
| | Variance | | ,000 | |
| | Std. Deviation | | ,0009858359 | |
| | Minimum | | -,003020961 | |
| | Maximum | | ,0033883472 | |
| | Range | | ,0064093083 | |
| | Interquartile Range | | ,0012731669 | |
| | Skewness | | -,063 | ,156 |
| | Kurtosis | | ,761 | ,310 |
| 33,34 | Mean | | ,0005107779 | ,0000589451 |
| | 95% Confidence Interval for Mean | Lower Bound | ,0003946717 | |
| | | Upper Bound | ,0006268840 | |
| | 5% Trimmed Mean | | ,0005028451 | |
| | Median | | ,0005306640 | |
| | Variance | | ,000 | |
| | Std. Deviation | | ,0009226367 | |
| | Minimum | | -,002460835 | |
| | Maximum | | ,0032758157 | |

## X AXIS Descriptives

| | | | Statistic | Std. Error |
|---|---|---|---|---|
| | Range | | ,0057366509 | |
| | Interquartile Range | | ,0012161436 | |
| | Skewness | | ,057 | ,156 |
| | Kurtosis | | ,499 | ,310 |
| 35,36 | Mean | | ,0005099598 | ,0000556038 |
| | 95% Confidence Interval for Mean | Lower Bound | ,0004004350 | |
| | | Upper Bound | ,0006194846 | |
| | 5% Trimmed Mean | | ,0005075355 | |
| | Median | | ,0005035220 | |
| | Variance | | ,000 | |
| | Std. Deviation | | ,0008703379 | |
| | Minimum | | -,002554899 | |
| | Maximum | | ,0033421806 | |
| | Range | | ,0058970795 | |
| | Interquartile Range | | ,0011433883 | |
| | Skewness | | ,016 | ,156 |
| | Kurtosis | | ,581 | ,310 |
| 37,38 | Mean | | ,0004126229 | ,0000462770 |
| | 95% Confidence Interval for Mean | Lower Bound | ,0003214695 | |
| | | Upper Bound | ,0005037763 | |
| | 5% Trimmed Mean | | ,0004095575 | |
| | Median | | ,0004056691 | |
| | Variance | | ,000 | |
| | Std. Deviation | | ,0007243498 | |
| | Minimum | | -,002258597 | |
| | Maximum | | ,0026842525 | |
| | Range | | ,0049428491 | |
| | Interquartile Range | | ,0009940134 | |
| | Skewness | | -,030 | ,156 |
| | Kurtosis | | ,329 | ,310 |
| 39,40 | Mean | | ,0002941511 | ,0000354241 |
| | 95% Confidence Interval for Mean | Lower Bound | ,0002243751 | |
| | | Upper Bound | ,0003639272 | |
| | 5% Trimmed Mean | | ,0002934700 | |
| | Median | | ,0002778220 | |
| | Variance | | ,000 | |
| | Std. Deviation | | ,0005544750 | |
| | Minimum | | -,001584039 | |

## X AXIS Descriptives

| | | | Statistic | Std. Error |
|---|---|---|---|---|
| | Maximum | | ,0018851274 | |
| | Range | | ,0034691662 | |
| | Interquartile Range | | ,0007555621 | |
| | Skewness | | -,008 | ,156 |
| | Kurtosis | | ,139 | ,310 |
| 41,42 | Mean | | ,0001382077 | ,0000202430 |
| | 95% Confidence Interval for Mean | Lower Bound | ,0000983343 | |
| | | Upper Bound | ,0001780810 | |
| | 5% Trimmed Mean | | ,0001432808 | |
| | Median | | ,0001402215 | |
| | Variance | | ,000 | |
| | Std. Deviation | | ,0003168533 | |
| | Minimum | | -,001189085 | |
| | Maximum | | ,0008735133 | |
| | Range | | ,0020625983 | |
| | Interquartile Range | | ,0004269287 | |
| | Skewness | | -,367 | ,156 |
| | Kurtosis | | ,821 | ,310 |
| 43,44 | Mean | | -,000227620 | ,0001068576 |
| | 95% Confidence Interval for Mean | Lower Bound | -,000438101 | |
| | | Upper Bound | -,000017139 | |
| | 5% Trimmed Mean | | -,000248089 | |
| | Median | | -,000391829 | |
| | Variance | | ,000 | |
| | Std. Deviation | | ,0016725866 | |
| | Minimum | | -,003994682 | |
| | Maximum | | ,0045002192 | |
| | Range | | ,0084949016 | |
| | Interquartile Range | | ,0025165858 | |
| | Skewness | | ,196 | ,156 |
| | Kurtosis | | -,406 | ,310 |
| 45,46 | Mean | | -,000249842 | ,0000860082 |
| | 95% Confidence Interval for Mean | Lower Bound | -,000419255 | |
| | | Upper Bound | -,000080428 | |
| | 5% Trimmed Mean | | -,000212150 | |
| | Median | | -,000220688 | |
| | Variance | | ,000 | |
| | Std. Deviation | | ,0013462420 | |

## X AXIS Descriptives

| | | | Statistic | Std. Error |
|---|---|---|---|---|
| | Minimum | | -,004528986 | |
| | Maximum | | ,0027614867 | |
| | Range | | ,0072904725 | |
| | Interquartile Range | | ,0017675688 | |
| | Skewness | | -,379 | ,156 |
| | Kurtosis | | ,130 | ,310 |
| 47,48 | Mean | | -,000240486 | ,0000813958 |
| | 95% Confidence Interval for Mean | Lower Bound | -,000400814 | |
| | | Upper Bound | -,000080158 | |
| | 5% Trimmed Mean | | -,000201586 | |
| | Median | | -,000201562 | |
| | Variance | | ,000 | |
| | Std. Deviation | | ,0012740453 | |
| | Minimum | | -,004265590 | |
| | Maximum | | ,0025691772 | |
| | Range | | ,0068347674 | |
| | Interquartile Range | | ,0016904216 | |
| | Skewness | | -,356 | ,156 |
| | Kurtosis | | ,027 | ,310 |
| 49,50 | Mean | | -,000131140 | ,0000729641 |
| | 95% Confidence Interval for Mean | Lower Bound | -,000274860 | |
| | | Upper Bound | ,0000125798 | |
| | 5% Trimmed Mean | | -,000131671 | |
| | Median | | -,000151029 | |
| | Variance | | ,000 | |
| | Std. Deviation | | ,0011420684 | |
| | Minimum | | -,003556603 | |
| | Maximum | | ,0029439335 | |
| | Range | | ,0065005368 | |
| | Interquartile Range | | ,0015399570 | |
| | Skewness | | -,030 | ,156 |
| | Kurtosis | | -,199 | ,310 |
| 51,52 | Mean | | ,0000275336 | ,0000716005 |
| | 95% Confidence Interval for Mean | Lower Bound | -,000113500 | |
| | | Upper Bound | ,0001685675 | |
| | 5% Trimmed Mean | | ,0000204484 | |
| | Median | | ,0000500568 | |
| | Variance | | ,000 | |

## X AXIS Descriptives

| | | | Statistic | Std. Error |
|---|---|---|---|---|
| | Std. Deviation | | ,0011207249 | |
| | Minimum | | -,003001613 | |
| | Maximum | | ,0042898205 | |
| | Range | | ,0072914334 | |
| | Interquartile Range | | ,0014562022 | |
| | Skewness | | ,138 | ,156 |
| | Kurtosis | | ,495 | ,310 |
| 53,54 | Mean | | ,0000435064 | ,0000689935 |
| | 95% Confidence Interval for Mean | Lower Bound | -,000092392 | |
| | | Upper Bound | ,0001794053 | |
| | 5% Trimmed Mean | | ,0000446780 | |
| | Median | | ,0000789240 | |
| | Variance | | ,000 | |
| | Std. Deviation | | ,0010799190 | |
| | Minimum | | -,003102189 | |
| | Maximum | | ,0044328180 | |
| | Range | | ,0075350072 | |
| | Interquartile Range | | ,0014607731 | |
| | Skewness | | ,072 | ,156 |
| | Kurtosis | | ,795 | ,310 |
| 55,56 | Mean | | -,000105380 | ,0000612265 |
| | 95% Confidence Interval for Mean | Lower Bound | -,000225979 | |
| | | Upper Bound | ,0000152204 | |
| | 5% Trimmed Mean | | -,000105584 | |
| | Median | | -,000073806 | |
| | Variance | | ,000 | |
| | Std. Deviation | | ,0009583463 | |
| | Minimum | | -,003042522 | |
| | Maximum | | ,0027612225 | |
| | Range | | ,0058037449 | |
| | Interquartile Range | | ,0011672172 | |
| | Skewness | | -,031 | ,156 |
| | Kurtosis | | ,226 | ,310 |
| 57,58 | Mean | | -,000223161 | ,0000766808 |
| | 95% Confidence Interval for Mean | Lower Bound | -,000374202 | |
| | | Upper Bound | -,000072120 | |
| | 5% Trimmed Mean | | -,000207368 | |
| | Median | | -,000207827 | |

## X AXIS Descriptives

| | | | Statistic | Std. Error |
|---|---|---|---|---|
| | Variance | | ,000 | |
| | Std. Deviation | | ,0012002451 | |
| | Minimum | | -,003639461 | |
| | Maximum | | ,0027072182 | |
| | Range | | ,0063466790 | |
| | Interquartile Range | | ,0014361590 | |
| | Skewness | | -,171 | ,156 |
| | Kurtosis | | ,127 | ,310 |
| 59,60 | Mean | | -,000269791 | ,0000871822 |
| | 95% Confidence Interval for Mean | Lower Bound | -,000441517 | |
| | | Upper Bound | -,000098066 | |
| | 5% Trimmed Mean | | -,000252051 | |
| | Median | | -,000296667 | |
| | Variance | | ,000 | |
| | Std. Deviation | | ,0013646165 | |
| | Minimum | | -,004166241 | |
| | Maximum | | ,0028322358 | |
| | Range | | ,0069984763 | |
| | Interquartile Range | | ,0017918683 | |
| | Skewness | | -,172 | ,156 |
| | Kurtosis | | -,071 | ,310 |
| 61,62 | Mean | | -,000220828 | ,0000639908 |
| | 95% Confidence Interval for Mean | Lower Bound | -,000346873 | |
| | | Upper Bound | -,000094783 | |
| | 5% Trimmed Mean | | -,000217334 | |
| | Median | | -,000210821 | |
| | Variance | | ,000 | |
| | Std. Deviation | | ,0010016151 | |
| | Minimum | | -,003054321 | |
| | Maximum | | ,0021735364 | |
| | Range | | ,0052278570 | |
| | Interquartile Range | | ,0013460837 | |
| | Skewness | | -,031 | ,156 |
| | Kurtosis | | -,238 | ,310 |
| 63,64 | Mean | | ,0000840169 | ,0000601878 |
| | 95% Confidence Interval for Mean | Lower Bound | -,000034537 | |
| | | Upper Bound | ,0002025709 | |
| | 5% Trimmed Mean | | ,0000825053 | |

## X AXIS Descriptives

| | | | Statistic | Std. Error |
|---|---|---|---|---|
| | Median | | ,0001039782 | |
| | Variance | | ,000 | |
| | Std. Deviation | | ,0009420885 | |
| | Minimum | | -,002526809 | |
| | Maximum | | ,0024284013 | |
| | Range | | ,0049552099 | |
| | Interquartile Range | | ,0013464582 | |
| | Skewness | | ,043 | ,156 |
| | Kurtosis | | -,238 | ,310 |
| 65,66 | Mean | | ,0002309626 | ,0000564059 |
| | 95% Confidence Interval for Mean | Lower Bound | ,0001198580 | |
| | | Upper Bound | ,0003420672 | |
| | 5% Trimmed Mean | | ,0002326745 | |
| | Median | | ,0002891808 | |
| | Variance | | ,000 | |
| | Std. Deviation | | ,0008828919 | |
| | Minimum | | -,002371352 | |
| | Maximum | | ,0026007027 | |
| | Range | | ,0049720546 | |
| | Interquartile Range | | ,0011540854 | |
| | Skewness | | -,075 | ,156 |
| | Kurtosis | | ,035 | ,310 |
| 67,68 | Mean | | ,0000808358 | ,0000464558 |
| | 95% Confidence Interval for Mean | Lower Bound | -,000010670 | |
| | | Upper Bound | ,0001723413 | |
| | 5% Trimmed Mean | | ,0000808804 | |
| | Median | | ,0001045974 | |
| | Variance | | ,000 | |
| | Std. Deviation | | ,0007271478 | |
| | Minimum | | -,001813749 | |
| | Maximum | | ,0021678370 | |
| | Range | | ,0039815858 | |
| | Interquartile Range | | ,0009062305 | |
| | Skewness | | ,051 | ,156 |
| | Kurtosis | | ,039 | ,310 |
| 69,70 | Mean | | -,000144087 | ,0000393539 |
| | 95% Confidence Interval for Mean | Lower Bound | -,000221604 | |
| | | Upper Bound | -,000066571 | |

## X AXIS Descriptives

| | | | Statistic | Std. Error |
|---|---|---|---|---|
| | 5% Trimmed Mean | | -,000146619 | |
| | Median | | -,000144633 | |
| | Variance | | ,000 | |
| | Std. Deviation | | ,0006159855 | |
| | Minimum | | -,002087320 | |
| | Maximum | | ,0018277232 | |
| | Range | | ,0039150431 | |
| | Interquartile Range | | ,0007945638 | |
| | Skewness | | -,019 | ,156 |
| | Kurtosis | | ,526 | ,310 |
| 71,72 | Mean | | ,0000221871 | ,0000468567 |
| | 95% Confidence Interval for Mean | Lower Bound | -,000070108 | |
| | | Upper Bound | ,0001144824 | |
| | 5% Trimmed Mean | | ,0000190753 | |
| | Median | | ,0000249080 | |
| | Variance | | ,000 | |
| | Std. Deviation | | ,0007334238 | |
| | Minimum | | -,002253232 | |
| | Maximum | | ,0021558484 | |
| | Range | | ,0044090799 | |
| | Interquartile Range | | ,0008999785 | |
| | Skewness | | -,020 | ,156 |
| | Kurtosis | | ,525 | ,310 |
| 73,74 | Mean | | ,0001005493 | ,0000535998 |
| | 95% Confidence Interval for Mean | Lower Bound | -,000005028 | |
| | | Upper Bound | ,0002061266 | |
| | 5% Trimmed Mean | | ,0001034964 | |
| | Median | | ,0001332430 | |
| | Variance | | ,000 | |
| | Std. Deviation | | ,0008389694 | |
| | Minimum | | -,002383079 | |
| | Maximum | | ,0025440950 | |
| | Range | | ,0049271735 | |
| | Interquartile Range | | ,0010222821 | |
| | Skewness | | -,071 | ,156 |
| | Kurtosis | | ,470 | ,310 |
| 75,76 | Mean | | -,000004876 | ,0000564025 |

## X AXIS Descriptives

| | | | Statistic | Std. Error |
|---|---|---|---|---|
| | 95% Confidence Interval for Mean | Lower Bound | -,000115974 | |
| | | Upper Bound | ,0001062220 | |
| | 5% Trimmed Mean | | ,0000001969 | |
| | Median | | ,0000165933 | |
| | Variance | | ,000 | |
| | Std. Deviation | | ,0008828390 | |
| | Minimum | | -,002710530 | |
| | Maximum | | ,0023856571 | |
| | Range | | ,0050961867 | |
| | Interquartile Range | | ,0010985329 | |
| | Skewness | | -,154 | ,156 |
| | Kurtosis | | ,255 | ,310 |
| 77,78 | Mean | | ,0002258512 | ,0000539680 |
| | 95% Confidence Interval for Mean | Lower Bound | ,0001195487 | |
| | | Upper Bound | ,0003321538 | |
| | 5% Trimmed Mean | | ,0002267454 | |
| | Median | | ,0002951020 | |
| | Variance | | ,000 | |
| | Std. Deviation | | ,0008447324 | |
| | Minimum | | -,002146211 | |
| | Maximum | | ,0027131992 | |
| | Range | | ,0048594107 | |
| | Interquartile Range | | ,0010189662 | |
| | Skewness | | -,004 | ,156 |
| | Kurtosis | | ,426 | ,310 |
| 79,80 | Mean | | ,0002258512 | ,0000539680 |
| | 95% Confidence Interval for Mean | Lower Bound | ,0001195487 | |
| | | Upper Bound | ,0003321538 | |
| | 5% Trimmed Mean | | ,0002267454 | |
| | Median | | ,0002951020 | |
| | Variance | | ,000 | |
| | Std. Deviation | | ,0008447324 | |
| | Minimum | | -,002146211 | |
| | Maximum | | ,0027131992 | |
| | Range | | ,0048594107 | |
| | Interquartile Range | | ,0010189662 | |
| | Skewness | | -,004 | ,156 |
| | Kurtosis | | ,426 | ,310 |

## X AXIS Descriptives

| | | | Statistic | Std. Error |
|---|---|---|---|---|
| 81,82 | Mean | | -,000055546 | ,0000188473 |
| | 95% Confidence Interval for Mean | Lower Bound | -,000092670 | |
| | | Upper Bound | -,000018422 | |
| | 5% Trimmed Mean | | -,000050972 | |
| | Median | | -,000049670 | |
| | Variance | | ,000 | |
| | Std. Deviation | | ,0002950070 | |
| | Minimum | | -,000945568 | |
| | Maximum | | ,0007068617 | |
| | Range | | ,0016524294 | |
| | Interquartile Range | | ,0003659813 | |
| | Skewness | | -,218 | ,156 |
| | Kurtosis | | ,320 | ,310 |
| 83,84 | Mean | | -,000184536 | ,0000215416 |
| | 95% Confidence Interval for Mean | Lower Bound | -,000226968 | |
| | | Upper Bound | -,000142105 | |
| | 5% Trimmed Mean | | -,000179556 | |
| | Median | | -,000145836 | |
| | Variance | | ,000 | |
| | Std. Deviation | | ,0003371792 | |
| | Minimum | | -,001174734 | |
| | Maximum | | ,0007306250 | |
| | Range | | ,0019053586 | |
| | Interquartile Range | | ,0004488236 | |
| | Skewness | | -,231 | ,156 |
| | Kurtosis | | ,053 | ,310 |
| 85,86 | Mean | | -,000220985 | ,0000296022 |
| | 95% Confidence Interval for Mean | Lower Bound | -,000279294 | |
| | | Upper Bound | -,000162677 | |
| | 5% Trimmed Mean | | -,000217546 | |
| | Median | | -,000180150 | |
| | Variance | | ,000 | |
| | Std. Deviation | | ,0004633479 | |
| | Minimum | | -,001540198 | |
| | Maximum | | ,0010661347 | |
| | Range | | ,0026063331 | |
| | Interquartile Range | | ,0006321438 | |
| | Skewness | | -,146 | ,156 |

## X AXIS Descriptives

| | | | Statistic | Std. Error |
|---|---|---|---|---|
| | Kurtosis | | ,061 | ,310 |
| 87,88 | Mean | | -,000184325 | ,0000203642 |
| | 95% Confidence Interval for Mean | Lower Bound | -,000224437 | |
| | | Upper Bound | -,000144213 | |
| | 5% Trimmed Mean | | -,000181947 | |
| | Median | | -,000172365 | |
| | Variance | | ,000 | |
| | Std. Deviation | | ,0003187497 | |
| | Minimum | | -,001153601 | |
| | Maximum | | ,0006871652 | |
| | Range | | ,0018407664 | |
| | Interquartile Range | | ,0004564656 | |
| | Skewness | | -,036 | ,156 |
| | Kurtosis | | -,200 | ,310 |
| 89,90 | Mean | | -,000141736 | ,0000155714 |
| | 95% Confidence Interval for Mean | Lower Bound | -,000172407 | |
| | | Upper Bound | -,000111064 | |
| | 5% Trimmed Mean | | -,000144390 | |
| | Median | | -,000149724 | |
| | Variance | | ,000 | |
| | Std. Deviation | | ,0002437317 | |
| | Minimum | | -,000799569 | |
| | Maximum | | ,0005082056 | |
| | Range | | ,0013077749 | |
| | Interquartile Range | | ,0003191285 | |
| | Skewness | | ,087 | ,156 |
| | Kurtosis | | -,112 | ,310 |
| 91,92 | Mean | | ,0002124531 | ,0000540864 |
| | 95% Confidence Interval for Mean | Lower Bound | ,0001059173 | |
| | | Upper Bound | ,0003189889 | |
| | 5% Trimmed Mean | | ,0002112602 | |
| | Median | | ,0002320276 | |
| | Variance | | ,000 | |
| | Std. Deviation | | ,0008465856 | |
| | Minimum | | -,002077043 | |
| | Maximum | | ,0026143419 | |
| | Range | | ,0046913850 | |
| | Interquartile Range | | ,0011741021 | |

### X AXIS Descriptives

| | | | Statistic | Std. Error |
|---|---|---|---|---|
| | Skewness | | ,035 | ,156 |
| | Kurtosis | | ,019 | ,310 |
| 93,94 | Mean | | ,0001298403 | ,0000341317 |
| | 95% Confidence Interval for Mean | Lower Bound | ,0000626099 | |
| | | Upper Bound | ,0001970708 | |
| | 5% Trimmed Mean | | ,0001280981 | |
| | Median | | ,0001401190 | |
| | Variance | | ,000 | |
| | Std. Deviation | | ,0005342460 | |
| | Minimum | | -,001368469 | |
| | Maximum | | ,0015462576 | |
| | Range | | ,0029147262 | |
| | Interquartile Range | | ,0007194778 | |
| | Skewness | | ,020 | ,156 |
| | Kurtosis | | ,034 | ,310 |
| 95,96 | Mean | | ,0001933900 | ,0000468171 |
| | 95% Confidence Interval for Mean | Lower Bound | ,0001011727 | |
| | | Upper Bound | ,0002856073 | |
| | 5% Trimmed Mean | | ,0001904234 | |
| | Median | | ,0001924188 | |
| | Variance | | ,000 | |
| | Std. Deviation | | ,0007328041 | |
| | Minimum | | -,001935712 | |
| | Maximum | | ,0022332070 | |
| | Range | | ,0041689191 | |
| | Interquartile Range | | ,0010029776 | |
| | Skewness | | ,063 | ,156 |
| | Kurtosis | | ,082 | ,310 |
| 97,98 | Mean | | ,0000457027 | ,0000141166 |
| | 95% Confidence Interval for Mean | Lower Bound | ,0000178967 | |
| | | Upper Bound | ,0000735086 | |
| | 5% Trimmed Mean | | ,0000454139 | |
| | Median | | ,0000590383 | |
| | Variance | | ,000 | |
| | Std. Deviation | | ,0002209595 | |
| | Minimum | | -,000607129 | |
| | Maximum | | ,0005995137 | |
| | Range | | ,0012066431 | |

## X AXIS Descriptives

| | | | Statistic | Std. Error |
|---|---|---|---|---|
| | Interquartile Range | | ,0003083711 | |
| | Skewness | | -,063 | ,156 |
| | Kurtosis | | -,215 | ,310 |
| 99,100 | Mean | | ,0000819929 | ,0000277677 |
| | 95% Confidence Interval for Mean | Lower Bound | ,0000272980 | |
| | | Upper Bound | ,0001366878 | |
| | 5% Trimmed Mean | | ,0000836778 | |
| | Median | | ,0000991283 | |
| | Variance | | ,000 | |
| | Std. Deviation | | ,0004346326 | |
| | Minimum | | -,001076990 | |
| | Maximum | | ,0012382431 | |
| | Range | | ,0023152327 | |
| | Interquartile Range | | ,0005594232 | |
| | Skewness | | -,074 | ,156 |
| | Kurtosis | | -,099 | ,310 |
| 101,102 | Mean | | ,0001412914 | ,0000399642 |
| | 95% Confidence Interval for Mean | Lower Bound | ,0000625725 | |
| | | Upper Bound | ,0002200103 | |
| | 5% Trimmed Mean | | ,0001415312 | |
| | Median | | ,0001714503 | |
| | Variance | | ,000 | |
| | Std. Deviation | | ,0006255390 | |
| | Minimum | | -,001771270 | |
| | Maximum | | ,0022756090 | |
| | Range | | ,0040468788 | |
| | Interquartile Range | | ,0007916464 | |
| | Skewness | | ,046 | ,156 |
| | Kurtosis | | ,396 | ,310 |
| 103,104 | Mean | | ,0000807301 | ,0000285315 |
| | 95% Confidence Interval for Mean | Lower Bound | ,0000245307 | |
| | | Upper Bound | ,0001369295 | |
| | 5% Trimmed Mean | | ,0000799971 | |
| | Median | | ,0000955327 | |
| | Variance | | ,000 | |
| | Std. Deviation | | ,0004465883 | |
| | Minimum | | -,001076992 | |
| | Maximum | | ,0012507966 | |

## X AXIS  Descriptives

| | | | Statistic | Std. Error |
|---|---|---|---|---|
| | Range | | ,0023277890 | |
| | Interquartile Range | | ,0006409496 | |
| | Skewness | | ,025 | ,156 |
| | Kurtosis | | -,238 | ,310 |
| 105,106 | Mean | | ,0000508855 | ,0000242428 |
| | 95% Confidence Interval for Mean | Lower Bound | ,0000031336 | |
| | | Upper Bound | ,0000986374 | |
| | 5% Trimmed Mean | | ,0000537412 | |
| | Median | | ,0000614648 | |
| | Variance | | ,000 | |
| | Std. Deviation | | ,0003794602 | |
| | Minimum | | -,000949343 | |
| | Maximum | | ,0011415339 | |
| | Range | | ,0020908773 | |
| | Interquartile Range | | ,0004971880 | |
| | Skewness | | -,048 | ,156 |
| | Kurtosis | | ,023 | ,310 |

## X AXIS Tests of Normality

| | Kolmogorov-Smirnov[a] | | | Shapiro-Wilk | | |
|---|---|---|---|---|---|---|
| | Statistic | df | Sig. | Statistic | df | Sig. |
| 11,12 | ,052 | 245 | ,200* | ,989 | 245 | ,056 |
| 13,14 | ,047 | 245 | ,200* | ,991 | 245 | ,134 |
| 15,16 | ,061 | 245 | ,028 | ,992 | 245 | ,196 |
| 17,18 | ,046 | 245 | ,200* | ,991 | 245 | ,146 |
| 19,20 | ,044 | 245 | ,200* | ,994 | 245 | ,463 |
| 21,22 | ,040 | 245 | ,200* | ,995 | 245 | ,526 |
| 23,24 | ,037 | 245 | ,200* | ,994 | 245 | ,514 |
| 25,26 | ,037 | 245 | ,200* | ,994 | 245 | ,434 |
| 27,28 | ,039 | 245 | ,200* | ,995 | 245 | ,616 |
| 29,30 | ,040 | 245 | ,200* | ,992 | 245 | ,231 |
| 31,32 | ,043 | 245 | ,200* | ,989 | 245 | ,071 |
| 33,34 | ,029 | 245 | ,200* | ,994 | 245 | ,478 |
| 35,36 | ,030 | 245 | ,200* | ,996 | 245 | ,821 |
| 37,38 | ,024 | 245 | ,200* | ,996 | 245 | ,731 |
| 39,40 | ,026 | 245 | ,200* | ,998 | 245 | ,989 |

## X AXIS Tests of Normality

| | Kolmogorov-Smirnov[a] | | | Shapiro-Wilk | | |
|---|---|---|---|---|---|---|
| | Statistic | df | Sig. | Statistic | df | Sig. |
| 41,42 | ,026 | 245 | ,200* | ,988 | 245 | ,037 |
| 43,44 | ,053 | 245 | ,093 | ,991 | 245 | ,112 |
| 45,46 | ,033 | 245 | ,200* | ,989 | 245 | ,052 |
| 47,48 | ,034 | 245 | ,200* | ,987 | 245 | ,031 |
| 49,50 | ,025 | 245 | ,200* | ,998 | 245 | ,993 |
| 51,52 | ,033 | 245 | ,200* | ,995 | 245 | ,556 |
| 53,54 | ,038 | 245 | ,200* | ,990 | 245 | ,094 |
| 55,56 | ,043 | 245 | ,200* | ,996 | 245 | ,775 |
| 57,58 | ,036 | 245 | ,200* | ,991 | 245 | ,157 |
| 59,60 | ,032 | 245 | ,200* | ,994 | 245 | ,472 |
| 61,62 | ,039 | 245 | ,200* | ,996 | 245 | ,785 |
| 63,64 | ,030 | 245 | ,200* | ,996 | 245 | ,830 |
| 65,66 | ,033 | 245 | ,200* | ,997 | 245 | ,921 |
| 67,68 | ,035 | 245 | ,200* | ,996 | 245 | ,764 |
| 69,70 | ,040 | 245 | ,200* | ,994 | 245 | ,494 |
| 71,72 | ,047 | 245 | ,200* | ,993 | 245 | ,321 |
| 73,74 | ,045 | 245 | ,200* | ,993 | 245 | ,280 |
| 75,76 | ,054 | 245 | ,081 | ,995 | 245 | ,581 |
| 77,78 | ,058 | 245 | ,046 | ,992 | 245 | ,229 |
| 79,80 | ,058 | 245 | ,046 | ,992 | 245 | ,229 |
| 81,82 | ,044 | 245 | ,200* | ,993 | 245 | ,252 |
| 83,84 | ,064 | 245 | ,016 | ,993 | 245 | ,306 |
| 85,86 | ,047 | 245 | ,200* | ,994 | 245 | ,431 |
| 87,88 | ,033 | 245 | ,200* | ,997 | 245 | ,888 |
| 89,90 | ,028 | 245 | ,200* | ,996 | 245 | ,753 |
| 91,92 | ,032 | 245 | ,200* | ,996 | 245 | ,860 |
| 93,94 | ,030 | 245 | ,200* | ,995 | 245 | ,627 |
| 95,96 | ,028 | 245 | ,200* | ,998 | 245 | ,985 |
| 97,98 | ,038 | 245 | ,200* | ,996 | 245 | ,833 |
| 99,100 | ,038 | 245 | ,200* | ,996 | 245 | ,756 |
| 101,102 | ,033 | 245 | ,200* | ,997 | 245 | ,939 |
| 103,104 | ,031 | 245 | ,200* | ,997 | 245 | ,954 |
| 105,106 | ,041 | 245 | ,200* | ,996 | 245 | ,739 |

*. This is a lower bound of the true significance.

a. Lilliefors Significance Correction

## Y AXIS  Descriptives

| | | | Statistic | Std. Error |
|---|---|---|---|---|
| 11,12 | Mean | | -,000244683 | ,0001247922 |
| | 95% Confidence Interval for Mean | Lower Bound | -,000490491 | |
| | | Upper Bound | ,0000011244 | |
| | 5% Trimmed Mean | | -,000244534 | |
| | Median | | -,000304924 | |
| | Variance | | ,000 | |
| | Std. Deviation | | ,0019533073 | |
| | Minimum | | -,005624473 | |
| | Maximum | | ,0059484379 | |
| | Range | | ,0115729113 | |
| | Interquartile Range | | ,0028445576 | |
| | Skewness | | ,053 | ,156 |
| | Kurtosis | | -,231 | ,310 |
| 13,14 | Mean | | -,000295515 | ,0001245327 |
| | 95% Confidence Interval for Mean | Lower Bound | -,000540812 | |
| | | Upper Bound | -,000050219 | |
| | 5% Trimmed Mean | | -,000292888 | |
| | Median | | -,000327816 | |
| | Variance | | ,000 | |
| | Std. Deviation | | ,0019492453 | |
| | Minimum | | -,005314051 | |
| | Maximum | | ,0056669056 | |
| | Range | | ,0109809567 | |
| | Interquartile Range | | ,0028267269 | |
| | Skewness | | ,057 | ,156 |
| | Kurtosis | | -,315 | ,310 |
| 15,16 | Mean | | -,000327807 | ,0001225270 |
| | 95% Confidence Interval for Mean | Lower Bound | -,000569152 | |
| | | Upper Bound | -,000086461 | |
| | 5% Trimmed Mean | | -,000330453 | |
| | Median | | -,000310285 | |
| | Variance | | ,000 | |
| | Std. Deviation | | ,0019178508 | |
| | Minimum | | -,005230925 | |
| | Maximum | | ,0056001269 | |
| | Range | | ,0108310518 | |
| | Interquartile Range | | ,0026623411 | |
| | Skewness | | ,066 | ,156 |

## Y AXIS Descriptives

| | | | Statistic | Std. Error |
|---|---|---|---|---|
| | Kurtosis | | -,316 | ,310 |
| 17,18 | Mean | | -,000359580 | ,0001214608 |
| | 95% Confidence Interval for Mean | Lower Bound | -,000598825 | |
| | | Upper Bound | -,000120334 | |
| | 5% Trimmed Mean | | -,000368113 | |
| | Median | | -,000413554 | |
| | Variance | | ,000 | |
| | Std. Deviation | | ,0019011616 | |
| | Minimum | | -,004884456 | |
| | Maximum | | ,0059847481 | |
| | Range | | ,0108692037 | |
| | Interquartile Range | | ,0026478201 | |
| | Skewness | | ,117 | ,156 |
| | Kurtosis | | -,200 | ,310 |
| 19,20 | Mean | | -,000334691 | ,0001214029 |
| | 95% Confidence Interval for Mean | Lower Bound | -,000573822 | |
| | | Upper Bound | -,000095559 | |
| | 5% Trimmed Mean | | -,000334626 | |
| | Median | | -,000354197 | |
| | Variance | | ,000 | |
| | Std. Deviation | | ,0019002560 | |
| | Minimum | | -,004848907 | |
| | Maximum | | ,0056704343 | |
| | Range | | ,0105193410 | |
| | Interquartile Range | | ,0026878314 | |
| | Skewness | | ,057 | ,156 |
| | Kurtosis | | -,286 | ,310 |
| 21,22 | Mean | | -,000284392 | ,0001214022 |
| | 95% Confidence Interval for Mean | Lower Bound | -,000523522 | |
| | | Upper Bound | -,000045262 | |
| | 5% Trimmed Mean | | -,000291795 | |
| | Median | | -,000330560 | |
| | Variance | | ,000 | |
| | Std. Deviation | | ,0019002448 | |
| | Minimum | | -,004484243 | |
| | Maximum | | ,0060552953 | |
| | Range | | ,0105395387 | |
| | Interquartile Range | | ,0027726032 | |

## Y AXIS Descriptives

| | | | Statistic | Std. Error |
|---|---|---|---|---|
| | Skewness | | ,121 | ,156 |
| | Kurtosis | | -,259 | ,310 |
| 23,24 | Mean | | -,000200313 | ,0001202109 |
| | 95% Confidence Interval for Mean | Lower Bound | -,000437096 | |
| | | Upper Bound | ,0000364705 | |
| | 5% Trimmed Mean | | -,000204088 | |
| | Median | | -,000229420 | |
| | Variance | | ,000 | |
| | Std. Deviation | | ,0018815982 | |
| | Minimum | | -,004811649 | |
| | Maximum | | ,0057631615 | |
| | Range | | ,0105748102 | |
| | Interquartile Range | | ,0025733468 | |
| | Skewness | | ,102 | ,156 |
| | Kurtosis | | -,189 | ,310 |
| 25,26 | Mean | | -,000083471 | ,0001151190 |
| | 95% Confidence Interval for Mean | Lower Bound | -,000310224 | |
| | | Upper Bound | ,0001432833 | |
| | 5% Trimmed Mean | | -,000095461 | |
| | Median | | -,000147025 | |
| | Variance | | ,000 | |
| | Std. Deviation | | ,0018018970 | |
| | Minimum | | -,004314668 | |
| | Maximum | | ,0052344175 | |
| | Range | | ,0095490860 | |
| | Interquartile Range | | ,0025607766 | |
| | Skewness | | ,131 | ,156 |
| | Kurtosis | | -,287 | ,310 |
| 27,28 | Mean | | ,0000161071 | ,0001060671 |
| | 95% Confidence Interval for Mean | Lower Bound | -,000192817 | |
| | | Upper Bound | ,0002250311 | |
| | 5% Trimmed Mean | | ,0000026116 | |
| | Median | | ,0000766338 | |
| | Variance | | ,000 | |
| | Std. Deviation | | ,0016602125 | |
| | Minimum | | -,003964513 | |
| | Maximum | | ,0051570260 | |
| | Range | | ,0091215386 | |

## Y AXIS  Descriptives

| | | | Statistic | Std. Error |
|---|---|---|---|---|
| | Interquartile Range | | ,0022683252 | |
| | Skewness | | ,145 | ,156 |
| | Kurtosis | | -,247 | ,310 |
| 29,30 | Mean | | ,0001288098 | ,0000931497 |
| | 95% Confidence Interval for Mean | Lower Bound | -,000054670 | |
| | | Upper Bound | ,0003122900 | |
| | 5% Trimmed Mean | | ,0001141128 | |
| | Median | | ,0001351496 | |
| | Variance | | ,000 | |
| | Std. Deviation | | ,0014580240 | |
| | Minimum | | -,003849334 | |
| | Maximum | | ,0043585721 | |
| | Range | | ,0082079064 | |
| | Interquartile Range | | ,0019292265 | |
| | Skewness | | ,167 | ,156 |
| | Kurtosis | | -,086 | ,310 |
| 31,32 | Mean | | ,0002022773 | ,0000788557 |
| | 95% Confidence Interval for Mean | Lower Bound | ,0000469525 | |
| | | Upper Bound | ,0003576022 | |
| | 5% Trimmed Mean | | ,0001910732 | |
| | Median | | ,0002409878 | |
| | Variance | | ,000 | |
| | Std. Deviation | | ,0012342874 | |
| | Minimum | | -,002777159 | |
| | Maximum | | ,0039002995 | |
| | Range | | ,0066774582 | |
| | Interquartile Range | | ,0015561870 | |
| | Skewness | | ,152 | ,156 |
| | Kurtosis | | -,033 | ,310 |
| 33,34 | Mean | | ,0002932439 | ,0000648629 |
| | 95% Confidence Interval for Mean | Lower Bound | ,0001654813 | |
| | | Upper Bound | ,0004210065 | |
| | 5% Trimmed Mean | | ,0002951889 | |
| | Median | | ,0002799194 | |
| | Variance | | ,000 | |
| | Std. Deviation | | ,0010152645 | |
| | Minimum | | -,002228756 | |
| | Maximum | | ,0034737345 | |

## Y AXIS  Descriptives

| | | | Statistic | Std. Error |
|---|---|---|---|---|
| | Range | | ,0057024907 | |
| | Interquartile Range | | ,0012232188 | |
| | Skewness | | ,025 | ,156 |
| | Kurtosis | | ,152 | ,310 |
| 35,36 | Mean | | ,0003036773 | ,0000560906 |
| | 95% Confidence Interval for Mean | Lower Bound | ,0001931937 | |
| | | Upper Bound | ,0004141609 | |
| | 5% Trimmed Mean | | ,0003096451 | |
| | Median | | ,0003425266 | |
| | Variance | | ,000 | |
| | Std. Deviation | | ,0008779571 | |
| | Minimum | | -,001991866 | |
| | Maximum | | ,0026422577 | |
| | Range | | ,0046341238 | |
| | Interquartile Range | | ,0011846316 | |
| | Skewness | | -,102 | ,156 |
| | Kurtosis | | -,250 | ,310 |
| 37,38 | Mean | | ,0003272164 | ,0000555361 |
| | 95% Confidence Interval for Mean | Lower Bound | ,0002178251 | |
| | | Upper Bound | ,0004366076 | |
| | 5% Trimmed Mean | | ,0003528120 | |
| | Median | | ,0003671443 | |
| | Variance | | ,000 | |
| | Std. Deviation | | ,0008692769 | |
| | Minimum | | -,002176287 | |
| | Maximum | | ,0021050400 | |
| | Range | | ,0042813268 | |
| | Interquartile Range | | ,0011584471 | |
| | Skewness | | -,397 | ,156 |
| | Kurtosis | | -,080 | ,310 |
| 39,40 | Mean | | ,0003276207 | ,0000622190 |
| | 95% Confidence Interval for Mean | Lower Bound | ,0002050659 | |
| | | Upper Bound | ,0004501756 | |
| | 5% Trimmed Mean | | ,0003439565 | |
| | Median | | ,0003994131 | |
| | Variance | | ,000 | |
| | Std. Deviation | | ,0009738810 | |
| | Minimum | | -,002487050 | |

## Y AXIS Descriptives

| | | | Statistic | Std. Error |
|---|---|---|---|---|
| | Maximum | | ,0031571762 | |
| | Range | | ,0056442261 | |
| | Interquartile Range | | ,0013050076 | |
| | Skewness | | -,293 | ,156 |
| | Kurtosis | | -,030 | ,310 |
| 41,42 | Mean | | ,0003674284 | ,0000734537 |
| | 95% Confidence Interval for Mean | Lower Bound | ,0002227441 | |
| | | Upper Bound | ,0005121128 | |
| | 5% Trimmed Mean | | ,0003880302 | |
| | Median | | ,0004368752 | |
| | Variance | | ,000 | |
| | Std. Deviation | | ,0011497330 | |
| | Minimum | | -,003291129 | |
| | Maximum | | ,0043469943 | |
| | Range | | ,0076381236 | |
| | Interquartile Range | | ,0014772339 | |
| | Skewness | | -,203 | ,156 |
| | Kurtosis | | ,246 | ,310 |
| 43,44 | Mean | | -,000237104 | ,0000807752 |
| | 95% Confidence Interval for Mean | Lower Bound | -,000396210 | |
| | | Upper Bound | -,000077999 | |
| | 5% Trimmed Mean | | -,000233175 | |
| | Median | | -,000246702 | |
| | Variance | | ,000 | |
| | Std. Deviation | | ,0012643326 | |
| | Minimum | | -,004529149 | |
| | Maximum | | ,0037124977 | |
| | Range | | ,0082416470 | |
| | Interquartile Range | | ,0017211066 | |
| | Skewness | | -,034 | ,156 |
| | Kurtosis | | ,387 | ,310 |
| 45,46 | Mean | | -,000185294 | ,0000777172 |
| | 95% Confidence Interval for Mean | Lower Bound | -,000338376 | |
| | | Upper Bound | -,000032212 | |
| | 5% Trimmed Mean | | -,000185410 | |
| | Median | | -,000250746 | |
| | Variance | | ,000 | |
| | Std. Deviation | | ,0012164666 | |

## Y AXIS Descriptives

| | | | Statistic | Std. Error |
|---|---|---|---|---|
| | Minimum | | -,003981504 | |
| | Maximum | | ,0041390910 | |
| | Range | | ,0081205951 | |
| | Interquartile Range | | ,0015075906 | |
| | Skewness | | ,063 | ,156 |
| | Kurtosis | | ,971 | ,310 |
| 47,48 | Mean | | -,000259171 | ,0000773953 |
| | 95% Confidence Interval for Mean | Lower Bound | -,000411619 | |
| | | Upper Bound | -,000106723 | |
| | 5% Trimmed Mean | | -,000264843 | |
| | Median | | -,000267945 | |
| | Variance | | ,000 | |
| | Std. Deviation | | ,0012114281 | |
| | Minimum | | -,003984324 | |
| | Maximum | | ,0031303196 | |
| | Range | | ,0071146431 | |
| | Interquartile Range | | ,0016129389 | |
| | Skewness | | ,060 | ,156 |
| | Kurtosis | | ,023 | ,310 |
| 49,50 | Mean | | -,000304502 | ,0000863651 |
| | 95% Confidence Interval for Mean | Lower Bound | -,000474619 | |
| | | Upper Bound | -,000134386 | |
| | 5% Trimmed Mean | | -,000311776 | |
| | Median | | -,000303258 | |
| | Variance | | ,000 | |
| | Std. Deviation | | ,0013518279 | |
| | Minimum | | -,004204592 | |
| | Maximum | | ,0033179714 | |
| | Range | | ,0075225633 | |
| | Interquartile Range | | ,0020692090 | |
| | Skewness | | -,017 | ,156 |
| | Kurtosis | | -,309 | ,310 |
| 51,52 | Mean | | -,000166892 | ,0000992228 |
| | 95% Confidence Interval for Mean | Lower Bound | -,000362334 | |
| | | Upper Bound | ,0000285506 | |
| | 5% Trimmed Mean | | -,000173390 | |
| | Median | | -,000221043 | |
| | Variance | | ,000 | |

## Y AXIS Descriptives

| | | | Statistic | Std. Error |
|---|---|---|---|---|
| | Std. Deviation | | ,0015530824 | |
| | Minimum | | -,004170230 | |
| | Maximum | | ,0040470415 | |
| | Range | | ,0082172719 | |
| | Interquartile Range | | ,0022172354 | |
| | Skewness | | -,003 | ,156 |
| | Kurtosis | | -,222 | ,310 |
| 53,54 | Mean | | -,000083900 | ,0000971946 |
| | 95% Confidence Interval for Mean | Lower Bound | -,000275348 | |
| | | Upper Bound | ,0001075473 | |
| | 5% Trimmed Mean | | -,000086800 | |
| | Median | | -,000140053 | |
| | Variance | | ,000 | |
| | Std. Deviation | | ,0015213364 | |
| | Minimum | | -,003865411 | |
| | Maximum | | ,0037977417 | |
| | Range | | ,0076631528 | |
| | Interquartile Range | | ,0021808381 | |
| | Skewness | | ,025 | ,156 |
| | Kurtosis | | -,256 | ,310 |
| 55,56 | Mean | | -,000189767 | ,0000821544 |
| | 95% Confidence Interval for Mean | Lower Bound | -,000351589 | |
| | | Upper Bound | -,000027944 | |
| | 5% Trimmed Mean | | -,000201396 | |
| | Median | | -,000185942 | |
| | Variance | | ,000 | |
| | Std. Deviation | | ,0012859205 | |
| | Minimum | | -,004109148 | |
| | Maximum | | ,0032802342 | |
| | Range | | ,0073893817 | |
| | Interquartile Range | | ,0018261882 | |
| | Skewness | | ,089 | ,156 |
| | Kurtosis | | -,184 | ,310 |
| 57,58 | Mean | | -,000195194 | ,0000721995 |
| | 95% Confidence Interval for Mean | Lower Bound | -,000337408 | |
| | | Upper Bound | -,000052981 | |
| | 5% Trimmed Mean | | -,000215092 | |
| | Median | | -,000286916 | |

## Y AXIS Descriptives

| | | | Statistic | Std. Error |
|---|---|---|---|---|
| | Variance | | ,000 | |
| | Std. Deviation | | ,0011301003 | |
| | Minimum | | -,003428758 | |
| | Maximum | | ,0033714833 | |
| | Range | | ,0068002415 | |
| | Interquartile Range | | ,0014769673 | |
| | Skewness | | ,225 | ,156 |
| | Kurtosis | | ,208 | ,310 |
| 59,60 | Mean | | -,000246237 | ,0000691215 |
| | 95% Confidence Interval for Mean | Lower Bound | -,000382388 | |
| | | Upper Bound | -,000110086 | |
| | 5% Trimmed Mean | | -,000251021 | |
| | Median | | -,000268283 | |
| | Variance | | ,000 | |
| | Std. Deviation | | ,0010819226 | |
| | Minimum | | -,003677678 | |
| | Maximum | | ,0034019818 | |
| | Range | | ,0070796594 | |
| | Interquartile Range | | ,0013419178 | |
| | Skewness | | ,069 | ,156 |
| | Kurtosis | | ,546 | ,310 |
| 61,62 | Mean | | ,0001321100 | ,0000553245 |
| | 95% Confidence Interval for Mean | Lower Bound | ,0000231354 | |
| | | Upper Bound | ,0002410846 | |
| | 5% Trimmed Mean | | ,0001306056 | |
| | Median | | ,0000686083 | |
| | Variance | | ,000 | |
| | Std. Deviation | | ,0008659658 | |
| | Minimum | | -,002846134 | |
| | Maximum | | ,0022856627 | |
| | Range | | ,0051317966 | |
| | Interquartile Range | | ,0012453910 | |
| | Skewness | | ,007 | ,156 |
| | Kurtosis | | ,110 | ,310 |
| 63,64 | Mean | | ,0001428781 | ,0000524419 |
| | 95% Confidence Interval for Mean | Lower Bound | ,0000395815 | |
| | | Upper Bound | ,0002461748 | |
| | 5% Trimmed Mean | | ,0001488662 | |

## Y AXIS  Descriptives

| | | | Statistic | Std. Error |
|---|---|---|---|---|
| | Median | | ,0002011775 | |
| | Variance | | ,000 | |
| | Std. Deviation | | ,0008208459 | |
| | Minimum | | -,002778163 | |
| | Maximum | | ,0020327061 | |
| | Range | | ,0048108695 | |
| | Interquartile Range | | ,0012640085 | |
| | Skewness | | -,168 | ,156 |
| | Kurtosis | | -,032 | ,310 |
| 65,66 | Mean | | ,0002019674 | ,0000495643 |
| | 95% Confidence Interval for Mean | Lower Bound | ,0001043388 | |
| | | Upper Bound | ,0002995959 | |
| | 5% Trimmed Mean | | ,0002117574 | |
| | Median | | ,0002675326 | |
| | Variance | | ,000 | |
| | Std. Deviation | | ,0007758045 | |
| | Minimum | | -,002391422 | |
| | Maximum | | ,0020628619 | |
| | Range | | ,0044542842 | |
| | Interquartile Range | | ,0011296647 | |
| | Skewness | | -,173 | ,156 |
| | Kurtosis | | -,041 | ,310 |
| 67,68 | Mean | | ,0001854673 | ,0000494553 |
| | 95% Confidence Interval for Mean | Lower Bound | ,0000880535 | |
| | | Upper Bound | ,0002828812 | |
| | 5% Trimmed Mean | | ,0001882861 | |
| | Median | | ,0002099886 | |
| | Variance | | ,000 | |
| | Std. Deviation | | ,0007740984 | |
| | Minimum | | -,001930642 | |
| | Maximum | | ,0022723149 | |
| | Range | | ,0042029564 | |
| | Interquartile Range | | ,0011576561 | |
| | Skewness | | -,020 | ,156 |
| | Kurtosis | | -,395 | ,310 |
| 69,70 | Mean | | ,0000238691 | ,0000530022 |
| | 95% Confidence Interval for Mean | Lower Bound | -,000080531 | |
| | | Upper Bound | ,0001282694 | |

## Y AXIS Descriptives

| | | | Statistic | Std. Error |
|---|---|---|---|---|
| | 5% Trimmed Mean | | ,0000219066 | |
| | Median | | ,0000497583 | |
| | Variance | | ,000 | |
| | Std. Deviation | | ,0008296155 | |
| | Minimum | | -,001769340 | |
| | Maximum | | ,0023747690 | |
| | Range | | ,0041441089 | |
| | Interquartile Range | | ,0011429362 | |
| | Skewness | | ,057 | ,156 |
| | Kurtosis | | -,410 | ,310 |
| 71,72 | Mean | | ,0000967947 | ,0000515778 |
| | 95% Confidence Interval for Mean | Lower Bound | -,000004800 | |
| | | Upper Bound | ,0001983892 | |
| | 5% Trimmed Mean | | ,0000995833 | |
| | Median | | ,0001453698 | |
| | Variance | | ,000 | |
| | Std. Deviation | | ,0008073204 | |
| | Minimum | | -,002180114 | |
| | Maximum | | ,0024696532 | |
| | Range | | ,0046497676 | |
| | Interquartile Range | | ,0011866402 | |
| | Skewness | | -,040 | ,156 |
| | Kurtosis | | -,318 | ,310 |
| 73,74 | Mean | | ,0001845866 | ,0000534973 |
| | 95% Confidence Interval for Mean | Lower Bound | ,0000792112 | |
| | | Upper Bound | ,0002899620 | |
| | 5% Trimmed Mean | | ,0001889585 | |
| | Median | | ,0002396267 | |
| | Variance | | ,000 | |
| | Std. Deviation | | ,0008373649 | |
| | Minimum | | -,002514079 | |
| | Maximum | | ,0023404309 | |
| | Range | | ,0048545096 | |
| | Interquartile Range | | ,0012808151 | |
| | Skewness | | -,117 | ,156 |
| | Kurtosis | | -,052 | ,310 |
| 75,76 | Mean | | ,0002182225 | ,0000559053 |

## Y AXIS Descriptives

| | | | Statistic | Std. Error |
|---|---|---|---|---|
| | 95% Confidence Interval for Mean | Lower Bound | ,0001081038 | |
| | | Upper Bound | ,0003283411 | |
| | 5% Trimmed Mean | | ,0002210880 | |
| | Median | | ,0001915000 | |
| | Variance | | ,000 | |
| | Std. Deviation | | ,0008750570 | |
| | Minimum | | -,002815487 | |
| | Maximum | | ,0023469415 | |
| | Range | | ,0051624287 | |
| | Interquartile Range | | ,0012291942 | |
| | Skewness | | -,113 | ,156 |
| | Kurtosis | | ,218 | ,310 |
| 77,78 | Mean | | ,0004086501 | ,0000597536 |
| | 95% Confidence Interval for Mean | Lower Bound | ,0002909514 | |
| | | Upper Bound | ,0005263488 | |
| | 5% Trimmed Mean | | ,0004066803 | |
| | Median | | ,0003806149 | |
| | Variance | | ,000 | |
| | Std. Deviation | | ,0009352915 | |
| | Minimum | | -,002775008 | |
| | Maximum | | ,0031084853 | |
| | Range | | ,0058834930 | |
| | Interquartile Range | | ,0012318402 | |
| | Skewness | | ,043 | ,156 |
| | Kurtosis | | ,250 | ,310 |
| 79,80 | Mean | | ,0004086501 | ,0000597536 |
| | 95% Confidence Interval for Mean | Lower Bound | ,0002909514 | |
| | | Upper Bound | ,0005263488 | |
| | 5% Trimmed Mean | | ,0004066803 | |
| | Median | | ,0003806149 | |
| | Variance | | ,000 | |
| | Std. Deviation | | ,0009352915 | |
| | Minimum | | -,002775008 | |
| | Maximum | | ,0031084853 | |
| | Range | | ,0058834930 | |
| | Interquartile Range | | ,0012318402 | |
| | Skewness | | ,043 | ,156 |
| | Kurtosis | | ,250 | ,310 |

## Y AXIS Descriptives

| | | | Statistic | Std. Error |
|---|---|---|---|---|
| 81,82 | Mean | | -,000190197 | ,0000646047 |
| | 95% Confidence Interval for Mean | Lower Bound | -,000317451 | |
| | | Upper Bound | -,000062943 | |
| | 5% Trimmed Mean | | -,000182528 | |
| | Median | | -,000202491 | |
| | Variance | | ,000 | |
| | Std. Deviation | | ,0010112228 | |
| | Minimum | | -,003099563 | |
| | Maximum | | ,0022167714 | |
| | Range | | ,0053163348 | |
| | Interquartile Range | | ,0013892032 | |
| | Skewness | | -,169 | ,156 |
| | Kurtosis | | -,138 | ,310 |
| 83,84 | Mean | | ,0001946568 | ,0000760509 |
| | 95% Confidence Interval for Mean | Lower Bound | ,0000448568 | |
| | | Upper Bound | ,0003444568 | |
| | 5% Trimmed Mean | | ,0001979172 | |
| | Median | | ,0002154575 | |
| | Variance | | ,000 | |
| | Std. Deviation | | ,0011903844 | |
| | Minimum | | -,003013756 | |
| | Maximum | | ,0032774352 | |
| | Range | | ,0062911916 | |
| | Interquartile Range | | ,0017002809 | |
| | Skewness | | -,038 | ,156 |
| | Kurtosis | | -,171 | ,310 |
| 85,86 | Mean | | ,0003148154 | ,0000730350 |
| | 95% Confidence Interval for Mean | Lower Bound | ,0001709559 | |
| | | Upper Bound | ,0004586749 | |
| | 5% Trimmed Mean | | ,0003162045 | |
| | Median | | ,0002602640 | |
| | Variance | | ,000 | |
| | Std. Deviation | | ,0011431784 | |
| | Minimum | | -,002717250 | |
| | Maximum | | ,0036521427 | |
| | Range | | ,0063693927 | |
| | Interquartile Range | | ,0016627308 | |
| | Skewness | | -,042 | ,156 |

## Y AXIS Descriptives

| | | | Statistic | Std. Error |
|---|---|---|---|---|
| | Kurtosis | | -,125 | ,310 |
| 87,88 | Mean | | ,0002925784 | ,0000850266 |
| | 95% Confidence Interval for Mean | Lower Bound | ,0001250987 | |
| | | Upper Bound | ,0004600582 | |
| | 5% Trimmed Mean | | ,0002943075 | |
| | Median | | ,0002701815 | |
| | Variance | | ,000 | |
| | Std. Deviation | | ,0013308764 | |
| | Minimum | | -,003151281 | |
| | Maximum | | ,0040445392 | |
| | Range | | ,0071958198 | |
| | Interquartile Range | | ,0020444959 | |
| | Skewness | | -,014 | ,156 |
| | Kurtosis | | -,373 | ,310 |
| 89,90 | Mean | | ,0002815765 | ,0001001301 |
| | 95% Confidence Interval for Mean | Lower Bound | ,0000843468 | |
| | | Upper Bound | ,0004788061 | |
| | 5% Trimmed Mean | | ,0002811619 | |
| | Median | | ,0003367688 | |
| | Variance | | ,000 | |
| | Std. Deviation | | ,0015672840 | |
| | Minimum | | -,003549944 | |
| | Maximum | | ,0043712062 | |
| | Range | | ,0079211497 | |
| | Interquartile Range | | ,0022294183 | |
| | Skewness | | -,034 | ,156 |
| | Kurtosis | | -,420 | ,310 |
| 91,92 | Mean | | ,0002322817 | ,0000875840 |
| | 95% Confidence Interval for Mean | Lower Bound | ,0000597645 | |
| | | Upper Bound | ,0004047989 | |
| | 5% Trimmed Mean | | ,0002282731 | |
| | Median | | ,0001142037 | |
| | Variance | | ,000 | |
| | Std. Deviation | | ,0013709063 | |
| | Minimum | | -,003990093 | |
| | Maximum | | ,0042777362 | |
| | Range | | ,0082678292 | |
| | Interquartile Range | | ,0018629280 | |

## Y AXIS Descriptives

| | | | Statistic | Std. Error |
|---|---|---|---|---|
| | Skewness | | ,046 | ,156 |
| | Kurtosis | | ,096 | ,310 |
| 93,94 | Mean | | -,000054863 | ,0000780323 |
| | 95% Confidence Interval for Mean | Lower Bound | -,000208566 | |
| | | Upper Bound | ,0000988399 | |
| | 5% Trimmed Mean | | -,000071438 | |
| | Median | | -,000131402 | |
| | Variance | | ,000 | |
| | Std. Deviation | | ,0012213986 | |
| | Minimum | | -,004164715 | |
| | Maximum | | ,0032298788 | |
| | Range | | ,0073945936 | |
| | Interquartile Range | | ,0017297109 | |
| | Skewness | | ,094 | ,156 |
| | Kurtosis | | ,371 | ,310 |
| 95,96 | Mean | | ,0001884256 | ,0000802886 |
| | 95% Confidence Interval for Mean | Lower Bound | ,0000302783 | |
| | | Upper Bound | ,0003465728 | |
| | 5% Trimmed Mean | | ,0001862152 | |
| | Median | | ,0001098661 | |
| | Variance | | ,000 | |
| | Std. Deviation | | ,0012567156 | |
| | Minimum | | -,004189587 | |
| | Maximum | | ,0038227344 | |
| | Range | | ,0080123219 | |
| | Interquartile Range | | ,0017045967 | |
| | Skewness | | -,012 | ,156 |
| | Kurtosis | | ,378 | ,310 |
| 97,98 | Mean | | -,000096210 | ,0000987215 |
| | 95% Confidence Interval for Mean | Lower Bound | -,000290666 | |
| | | Upper Bound | ,0000982447 | |
| | 5% Trimmed Mean | | -,000099723 | |
| | Median | | -,000135114 | |
| | Variance | | ,000 | |
| | Std. Deviation | | ,0015452364 | |
| | Minimum | | -,004349089 | |
| | Maximum | | ,0037616077 | |
| | Range | | ,0081106971 | |

## Y AXIS Descriptives

| | | | Statistic | Std. Error |
|---|---|---|---|---|
| | Interquartile Range | | ,0021809197 | |
| | Skewness | | ,053 | ,156 |
| | Kurtosis | | -,206 | ,310 |
| 99,100 | Mean | | -,000079174 | ,0000766426 |
| | 95% Confidence Interval for Mean | Lower Bound | -,000230140 | |
| | | Upper Bound | ,0000717915 | |
| | 5% Trimmed Mean | | -,000086544 | |
| | Median | | -,000125995 | |
| | Variance | | ,000 | |
| | Std. Deviation | | ,0011996464 | |
| | Minimum | | -,004777641 | |
| | Maximum | | ,0031041802 | |
| | Range | | ,0078818211 | |
| | Interquartile Range | | ,0015197809 | |
| | Skewness | | -,046 | ,156 |
| | Kurtosis | | ,764 | ,310 |
| 101,102 | Mean | | -,000096588 | ,0000790601 |
| | 95% Confidence Interval for Mean | Lower Bound | -,000252315 | |
| | | Upper Bound | ,0000591397 | |
| | 5% Trimmed Mean | | -,000099303 | |
| | Median | | -,000136496 | |
| | Variance | | ,000 | |
| | Std. Deviation | | ,0012374867 | |
| | Minimum | | -,004611117 | |
| | Maximum | | ,0034179833 | |
| | Range | | ,0080291005 | |
| | Interquartile Range | | ,0015491906 | |
| | Skewness | | ,013 | ,156 |
| | Kurtosis | | ,734 | ,310 |
| 103,104 | Mean | | -,000077199 | ,0000789636 |
| | 95% Confidence Interval for Mean | Lower Bound | -,000232736 | |
| | | Upper Bound | ,0000783380 | |
| | 5% Trimmed Mean | | -,000080144 | |
| | Median | | -,000072547 | |
| | Variance | | ,000 | |
| | Std. Deviation | | ,0012359752 | |
| | Minimum | | -,004995261 | |
| | Maximum | | ,0033251570 | |

## Y AXIS Descriptives

| | | | Statistic | Std. Error |
|---|---|---|---|---|
| | Range | | ,0083204183 | |
| | Interquartile Range | | ,0016687605 | |
| | Skewness | | -,084 | ,156 |
| | Kurtosis | | ,866 | ,310 |
| 105,106 | Mean | | -,000306255 | ,0000888729 |
| | 95% Confidence Interval for Mean | Lower Bound | -,000481311 | |
| | | Upper Bound | -,000131199 | |
| | 5% Trimmed Mean | | -,000309070 | |
| | Median | | -,000327404 | |
| | Variance | | ,000 | |
| | Std. Deviation | | ,0013910814 | |
| | Minimum | | -,005622719 | |
| | Maximum | | ,0035351289 | |
| | Range | | ,0091578480 | |
| | Interquartile Range | | ,0018152810 | |
| | Skewness | | -,074 | ,156 |
| | Kurtosis | | ,833 | ,310 |

## Y AXIS Tests of Normality

| | Kolmogorov-Smirnov[a] | | | Shapiro-Wilk | | |
|---|---|---|---|---|---|---|
| | Statistic | df | Sig. | Statistic | df | Sig. |
| 11,12 | ,043 | 245 | ,200* | ,996 | 245 | ,815 |
| 13,14 | ,036 | 245 | ,200* | ,996 | 245 | ,712 |
| 15,16 | ,038 | 245 | ,200* | ,996 | 245 | ,734 |
| 17,18 | ,029 | 245 | ,200* | ,996 | 245 | ,698 |
| 19,20 | ,031 | 245 | ,200* | ,996 | 245 | ,704 |
| 21,22 | ,055 | 245 | ,068 | ,993 | 245 | ,325 |
| 23,24 | ,048 | 245 | ,200* | ,996 | 245 | ,788 |
| 25,26 | ,066 | 245 | ,012 | ,994 | 245 | ,464 |
| 27,28 | ,064 | 245 | ,017 | ,994 | 245 | ,396 |
| 29,30 | ,037 | 245 | ,200* | ,996 | 245 | ,794 |
| 31,32 | ,036 | 245 | ,200* | ,996 | 245 | ,752 |
| 33,34 | ,036 | 245 | ,200* | ,995 | 245 | ,536 |
| 35,36 | ,023 | 245 | ,200* | ,996 | 245 | ,771 |
| 37,38 | ,064 | 245 | ,018 | ,986 | 245 | ,014 |
| 39,40 | ,045 | 245 | ,200* | ,991 | 245 | ,144 |

## Y AXIS Tests of Normality

| | Kolmogorov-Smirnov[a] | | | Shapiro-Wilk | | |
|---|---|---|---|---|---|---|
| | Statistic | df | Sig. | Statistic | df | Sig. |
| 41,42 | ,037 | 245 | ,200* | ,993 | 245 | ,290 |
| 43,44 | ,028 | 245 | ,200* | ,997 | 245 | ,873 |
| 45,46 | ,046 | 245 | ,200* | ,988 | 245 | ,045 |
| 47,48 | ,021 | 245 | ,200* | ,998 | 245 | ,983 |
| 49,50 | ,045 | 245 | ,200* | ,994 | 245 | ,514 |
| 51,52 | ,047 | 245 | ,200* | ,995 | 245 | ,648 |
| 53,54 | ,041 | 245 | ,200* | ,996 | 245 | ,704 |
| 55,56 | ,036 | 245 | ,200* | ,994 | 245 | ,433 |
| 57,58 | ,042 | 245 | ,200* | ,993 | 245 | ,342 |
| 59,60 | ,036 | 245 | ,200* | ,996 | 245 | ,771 |
| 61,62 | ,042 | 245 | ,200* | ,990 | 245 | ,073 |
| 63,64 | ,053 | 245 | ,092 | ,988 | 245 | ,046 |
| 65,66 | ,053 | 245 | ,087 | ,991 | 245 | ,163 |
| 67,68 | ,045 | 245 | ,200* | ,994 | 245 | ,495 |
| 69,70 | ,038 | 245 | ,200* | ,993 | 245 | ,358 |
| 71,72 | ,049 | 245 | ,200* | ,995 | 245 | ,647 |
| 73,74 | ,052 | 245 | ,200* | ,991 | 245 | ,154 |
| 75,76 | ,035 | 245 | ,200* | ,993 | 245 | ,316 |
| 77,78 | ,039 | 245 | ,200* | ,995 | 245 | ,569 |
| 79,80 | ,039 | 245 | ,200* | ,995 | 245 | ,569 |
| 81,82 | ,049 | 245 | ,200* | ,995 | 245 | ,571 |
| 83,84 | ,029 | 245 | ,200* | ,997 | 245 | ,916 |
| 85,86 | ,040 | 245 | ,200* | ,996 | 245 | ,852 |
| 87,88 | ,048 | 245 | ,200* | ,996 | 245 | ,783 |
| 89,90 | ,046 | 245 | ,200* | ,994 | 245 | ,446 |
| 91,92 | ,045 | 245 | ,200* | ,996 | 245 | ,821 |
| 93,94 | ,042 | 245 | ,200* | ,990 | 245 | ,081 |
| 95,96 | ,039 | 245 | ,200* | ,995 | 245 | ,648 |
| 97,98 | ,039 | 245 | ,200* | ,995 | 245 | ,609 |
| 99,100 | ,044 | 245 | ,200* | ,989 | 245 | ,062 |
| 101,102 | ,043 | 245 | ,200* | ,992 | 245 | ,213 |
| 103,104 | ,040 | 245 | ,200* | ,990 | 245 | ,107 |
| 105,106 | ,036 | 245 | ,200* | ,992 | 245 | ,177 |

*. This is a lower bound of the true significance.

a. Lilliefors Significance Correction

## X AXIS One-Sample Test

| | Test Value = 0 | | | | | |
|---|---|---|---|---|---|---|
| | | | | | 95% Confidence Interval of the Difference | |
| | t | df | Sig. (2-tailed) | Mean Difference | Lower | Upper |
| 11,12 | 3,972 | 244 | ,000 | ,0002313175 | ,0001166144 | ,0003460205 |
| 13,14 | 3,568 | 244 | ,000 | ,0001915536 | ,0000858075 | ,0002972997 |
| 15,16 | 2,924 | 244 | ,004 | ,0001462272 | ,0000477379 | ,0002447164 |
| 17,18 | 2,591 | 244 | ,010 | ,0001248311 | ,0000299213 | ,0002197409 |
| 19,20 | 2,748 | 244 | ,006 | ,0001310097 | ,0000371176 | ,0002249017 |
| 21,22 | 2,842 | 244 | ,005 | ,0001433855 | ,0000439941 | ,0002427770 |
| 23,24 | 3,911 | 244 | ,000 | ,0002096760 | ,0001040750 | ,0003152771 |
| 25,26 | 4,975 | 244 | ,000 | ,0002945792 | ,0001779358 | ,0004112225 |
| 27,28 | 5,820 | 244 | ,000 | ,0003601511 | ,0002382528 | ,0004820493 |
| 29,30 | 6,797 | 244 | ,000 | ,0004260654 | ,0003025998 | ,0005495309 |
| 31,32 | 7,966 | 244 | ,000 | ,0005016958 | ,0003776365 | ,0006257550 |
| 33,34 | 8,665 | 244 | ,000 | ,0005107779 | ,0003946717 | ,0006268840 |
| 35,36 | 9,171 | 244 | ,000 | ,0005099598 | ,0004004350 | ,0006194846 |
| 37,38 | 8,916 | 244 | ,000 | ,0004126229 | ,0003214695 | ,0005037763 |
| 39,40 | 8,304 | 244 | ,000 | ,0002941511 | ,0002243751 | ,0003639272 |
| 41,42 | 6,827 | 244 | ,000 | ,0001382077 | ,0000983343 | ,0001780810 |
| 43,44 | -2,130 | 244 | ,034 | -,000227620 | -,000438101 | -,000017139 |
| 45,46 | -2,905 | 244 | ,004 | -,000249842 | -,000419255 | -,000080428 |
| 47,48 | -2,955 | 244 | ,003 | -,000240486 | -,000400814 | -,000080158 |
| 49,50 | -1,797 | 244 | ,074 | -,000131140 | -,000274860 | ,0000125798 |
| 51,52 | ,385 | 244 | ,701 | ,0000275336 | -,000113500 | ,0001685675 |
| 53,54 | ,631 | 244 | ,529 | ,0000435064 | -,000092392 | ,0001794053 |
| 55,56 | -1,721 | 244 | ,086 | -,000105380 | -,000225979 | ,0000152204 |
| 57,58 | -2,910 | 244 | ,004 | -,000223161 | -,000374202 | -,000072120 |
| 59,60 | -3,095 | 244 | ,002 | -,000269791 | -,000441517 | -,000098066 |
| 61,62 | -3,451 | 244 | ,001 | -,000220828 | -,000346873 | -,000094783 |
| 63,64 | 1,396 | 244 | ,164 | ,0000840169 | -,000034537 | ,0002025709 |
| 65,66 | 4,095 | 244 | ,000 | ,0002309626 | ,0001198580 | ,0003420672 |
| 67,68 | 1,740 | 244 | ,083 | ,0000808358 | -,000010670 | ,0001723413 |
| 69,70 | -3,661 | 244 | ,000 | -,000144087 | -,000221604 | -,000066571 |
| 71,72 | ,474 | 244 | ,636 | ,0000221871 | -,000070108 | ,0001144824 |
| 73,74 | 1,876 | 244 | ,062 | ,0001005493 | -,000005028 | ,0002061266 |
| 75,76 | -,086 | 244 | ,931 | -,000004876 | -,000115974 | ,0001062220 |
| 77,78 | 4,185 | 244 | ,000 | ,0002258512 | ,0001195487 | ,0003321538 |
| 79,80 | 4,185 | 244 | ,000 | ,0002258512 | ,0001195487 | ,0003321538 |

## X AXIS  One-Sample Test

| | Test Value = 0 | | | | | |
|---|---|---|---|---|---|---|
| | | | | | 95% Confidence Interval of the Difference | |
| | t | df | Sig. (2-tailed) | Mean Difference | Lower | Upper |
| 81,82 | -2,947 | 244 | ,004 | -,000055546 | -,000092670 | -,000018422 |
| 83,84 | -8,567 | 244 | ,000 | -,000184536 | -,000226968 | -,000142105 |
| 85,86 | -7,465 | 244 | ,000 | -,000220985 | -,000279294 | -,000162677 |
| 87,88 | -9,051 | 244 | ,000 | -,000184325 | -,000224437 | -,000144213 |
| 89,90 | -9,102 | 244 | ,000 | -,000141736 | -,000172407 | -,000111064 |
| 91,92 | 3,928 | 244 | ,000 | ,0002124531 | ,0001059173 | ,0003189889 |
| 93,94 | 3,804 | 244 | ,000 | ,0001298403 | ,0000626099 | ,0001970708 |
| 95,96 | 4,131 | 244 | ,000 | ,0001933900 | ,0001011727 | ,0002856073 |
| 97,98 | 3,238 | 244 | ,001 | ,0000457027 | ,0000178967 | ,0000735086 |
| 99,100 | 2,953 | 244 | ,003 | ,0000819929 | ,0000272980 | ,0001366878 |
| 101,102 | 3,535 | 244 | ,000 | ,0001412914 | ,0000625725 | ,0002200103 |
| 103,104 | 2,830 | 244 | ,005 | ,0000807301 | ,0000245307 | ,0001369295 |
| 105,106 | 2,099 | 244 | ,037 | ,0000508855 | ,0000031336 | ,0000986374 |

## Y AXIS One-Sample Test

| | Test Value = 0 | | | | | |
|---|---|---|---|---|---|---|
| | | | | | 95% Confidence Interval of the Difference | |
| | t | df | Sig. (2-tailed) | Mean Difference | Lower | Upper |
| 11,12 | -1,961 | 244 | ,051 | -,000244683 | -,000490491 | ,0000011244 |
| 13,14 | -2,373 | 244 | ,018 | -,000295515 | -,000540812 | -,000050219 |
| 15,16 | -2,675 | 244 | ,008 | -,000327807 | -,000569152 | -,000086461 |
| 17,18 | -2,960 | 244 | ,003 | -,000359580 | -,000598825 | -,000120334 |
| 19,20 | -2,757 | 244 | ,006 | -,000334691 | -,000573822 | -,000095559 |
| 21,22 | -2,343 | 244 | ,020 | -,000284392 | -,000523522 | -,000045262 |
| 23,24 | -1,666 | 244 | ,097 | -,000200313 | -,000437096 | ,0000364705 |
| 25,26 | -,725 | 244 | ,469 | -,000083471 | -,000310224 | ,0001432833 |
| 27,28 | ,152 | 244 | ,879 | ,0000161071 | -,000192817 | ,0002250311 |
| 29,30 | 1,383 | 244 | ,168 | ,0001288098 | -,000054670 | ,0003122900 |
| 31,32 | 2,565 | 244 | ,011 | ,0002022773 | ,0000469525 | ,0003576022 |
| 33,34 | 4,521 | 244 | ,000 | ,0002932439 | ,0001654813 | ,0004210065 |
| 35,36 | 5,414 | 244 | ,000 | ,0003036773 | ,0001931937 | ,0004141609 |
| 37,38 | 5,892 | 244 | ,000 | ,0003272164 | ,0002178251 | ,0004366076 |
| 39,40 | 5,266 | 244 | ,000 | ,0003276207 | ,0002050659 | ,0004501756 |
| 41,42 | 5,002 | 244 | ,000 | ,0003674284 | ,0002227441 | ,0005121128 |

## Y AXIS One-Sample Test

| | Test Value = 0 | | | | | |
|---|---|---|---|---|---|---|
| | | | | | 95% Confidence Interval of the Difference | |
| | t | df | Sig. (2-tailed) | Mean Difference | Lower | Upper |
| 43,44 | -2,935 | 244 | ,004 | -,000237104 | -,000396210 | -,000077999 |
| 45,46 | -2,384 | 244 | ,018 | -,000185294 | -,000338376 | -,000032212 |
| 47,48 | -3,349 | 244 | ,001 | -,000259171 | -,000411619 | -,000106723 |
| 49,50 | -3,526 | 244 | ,001 | -,000304502 | -,000474619 | -,000134386 |
| 51,52 | -1,682 | 244 | ,094 | -,000166892 | -,000362334 | ,0000285506 |
| 53,54 | -,863 | 244 | ,389 | -,000083900 | -,000275348 | ,0001075473 |
| 55,56 | -2,310 | 244 | ,022 | -,000189767 | -,000351589 | -,000027944 |
| 57,58 | -2,704 | 244 | ,007 | -,000195194 | -,000337408 | -,000052981 |
| 59,60 | -3,562 | 244 | ,000 | -,000246237 | -,000382388 | -,000110086 |
| 61,62 | 2,388 | 244 | ,018 | ,0001321100 | ,0000231354 | ,0002410846 |
| 63,64 | 2,725 | 244 | ,007 | ,0001428781 | ,0000395815 | ,0002461748 |
| 65,66 | 4,075 | 244 | ,000 | ,0002019674 | ,0001043388 | ,0002995959 |
| 67,68 | 3,750 | 244 | ,000 | ,0001854673 | ,0000880535 | ,0002828812 |
| 69,70 | ,450 | 244 | ,653 | ,0000238691 | -,000080531 | ,0001282694 |
| 71,72 | 1,877 | 244 | ,062 | ,0000967947 | -,000004800 | ,0001983892 |
| 73,74 | 3,450 | 244 | ,001 | ,0001845866 | ,0000792112 | ,0002899620 |
| 75,76 | 3,903 | 244 | ,000 | ,0002182225 | ,0001081038 | ,0003283411 |
| 77,78 | 6,839 | 244 | ,000 | ,0004086501 | ,0002909514 | ,0005263488 |
| 79,80 | 6,839 | 244 | ,000 | ,0004086501 | ,0002909514 | ,0005263488 |
| 81,82 | -2,944 | 244 | ,004 | -,000190197 | -,000317451 | -,000062943 |
| 83,84 | 2,560 | 244 | ,011 | ,0001946568 | ,0000448568 | ,0003444568 |
| 85,86 | 4,310 | 244 | ,000 | ,0003148154 | ,0001709559 | ,0004586749 |
| 87,88 | 3,441 | 244 | ,001 | ,0002925784 | ,0001250987 | ,0004600582 |
| 89,90 | 2,812 | 244 | ,005 | ,0002815765 | ,0000843468 | ,0004788061 |
| 91,92 | 2,652 | 244 | ,009 | ,0002322817 | ,0000597645 | ,0004047989 |
| 93,94 | -,703 | 244 | ,483 | -,000054863 | -,000208566 | ,0000988399 |
| 95,96 | 2,347 | 244 | ,020 | ,0001884256 | ,0000302783 | ,0003465728 |
| 97,98 | -,975 | 244 | ,331 | -,000096210 | -,000290666 | ,0000982447 |
| 99,100 | -1,033 | 244 | ,303 | -,000079174 | -,000230140 | ,0000717915 |
| 101,102 | -1,222 | 244 | ,223 | -,000096588 | -,000252315 | ,0000591397 |
| 103,104 | -,978 | 244 | ,329 | -,000077199 | -,000232736 | ,0000783380 |
| 105,106 | -3,446 | 244 | ,001 | -,000306255 | -,000481311 | -,000131199 |
