# Supplementary material for: Testing strategic pluralism: The roles of attractiveness and competitive abilities to understand conditionality in men’s short-term reproductive strategies
Source: PLoS One. 2020 Aug 31;15(8):e0237315. doi: 10.1371/journal.pone.0237315 (PMC7458284; doi:10.1371/journal.pone.0237315)
Supplement: Attachment — Submitted filename: PONE-D-19-27652_review.pdf [file pone.0237315.s003.pdf]

PONE-D-19-27652
Testing strategic pluralism: The roles of attractiveness and competitive abilities to understand conditionality in men's short-term reproductive strategies

Oriana Figueroa, José Antonio Muñoz-Reyes, Carlos Rodriguez-Sickert, Nohelia Valenzuela, Paula Pavez, Oriana Ramírez-Herrera, Miguel Pita, David Diaz, Ana Fernández, Pablo Polo

This study gathers together evidence in support of the Strategic Pluralism Hypothesis, the idea that males can allocate time and resources to either mate selection or the raising of children. The study is well done and believable, but the paper can be improved especially in the presentation of the Strategic Pluralism Hypothesis and its predictions.

The Strategic Pluralism Hypothesis is presented first, followed by a set of the sub-hypotheses. These sub-hypotheses are actually predictions of the Strategic Pluralism Hypothesis, but is unclear how they are related. They also need to be portrayed as predictions. For example, if SPH is true, then we expect *a*, *b*, and *c* to be true. Finally, what are the alternatives to the SPH? Science functions best when there are alternative explanations for phenomena.

The Strategic Pluralism Hypothesis is posed as an either/or affair, when it is really an entire spectrum. The two ends of the spectrum are 100% of the time and energy spent trying to mate with every available partner versus 100% of the time spent raising children with one partner. But the time and energy allocations might be 50/50 or 70/30. If the optimal allocations for males are 50/50, how would that effect the predictions? Would one be able to detect an effect? And although it isn't mentioned in the text, sexually transmitted diseases might enter into the balance of selective forces as well.

Facial symmetry is employed as an indicator of facial attractiveness. The authors portray this symmetry/asymmetry as fluctuating asymmetry. But fluctuating asymmetry is a population parameter. Individual asymmetry may collectively represent fluctuating asymmetry, or it may represent directional asymmetry or antisymmetry or a mixture of all three kinds of asymmetry. The authors mention directional asymmetry when they mention the morphometric analysis and Procrustes distance, but that is the last time it appears. Was there detectable directional asymmetry?

The other two forms of asymmetry (directional asymmetry and antisymmetry) may be better indicators of male quality than fluctuating asymmetry. If low fitness males exhibit fluctuating asymmetry, the modal low fitness males will still be perfectly symmetrical. But if low fitness is associated with a transition from fluctuating asymmetry to directional asymmetry (or antisymmetry), then the predictive value of individual asymmetry is much better. There is a literature for this and evidence for transitions among the three forms of asymmetry in populations.

Throughout the text, the authors mention that fluctuating facial asymmetry is associated with attractiveness. This is a misleading way to portray this association, because it is symmetry, not asymmetry, that is considered attractive. Moreover, it is "individual" symmetry (or asymmetry)

that potential mates respond to. Fluctuating asymmetry is assumed to underlie the variation in individual asymmetries. This is an assumption that must be tested, but it rarely is. At the very least it needs to be mentioned.

In addition, the evidence that fluctuating asymmetry is an indicator of genetic quality has been long discussed, and without resolution. It would pay to study (and cite) some of the papers by population geneticists. They generally aren't sympathetic to the idea of "genetic" quality. How would you measure it? In what sense might a male have "quality" genes? This brings us to Darwinian and inclusive fitness. There are very few studies that have actually looked at fluctuating asymmetry and all possible fitness components (ability to find a mate, fertility, etc.).

For the measurement of fluctuating asymmetry, what is the measurement error associated with the approach? All studies of FA need to assess measurement error, because it can inflate estimates of FA. To do this, the authors need to take more than one photo of each person and then use FACE ++ and MorphoJ to estimate overall asymmetry and follow that with a variance component ANOVA. The methodology in this section is extremely unclear. I presume that a single, unitless number is produced (Procrustes distance), with larger numbers indicating more asymmetry.

For the statistical analysis, the relationship between the Strategic Pluralism Hypothesis and its predictions is unclear. You need to make clear, for example, that the SPH predicts a relationship between socio-sexual orientation and perception of attractiveness and facial symmetry. Moreover, there are no alternative hypotheses mentioned in the paper. If the results didn't support SPH, what would they support?

The English is grammatically good, though somewhat wordy. In several places, I found the meaning of a phrase or sentence to be obscure. This is usually attributable to wordiness and passive voice.

In the figures, it would be more effective to spell out SOI, FFA, and SPA. I was finding myself having to look these up every time I came across them.

I have also included many comments on the manuscript itself.
