# Supplementary material for: Testing strategic pluralism: The roles of attractiveness and competitive abilities to understand conditionality in men’s short-term reproductive strategies
Source: PLoS One. 2020 Aug 31;15(8):e0237315. doi: 10.1371/journal.pone.0237315 (PMC7458284; doi:10.1371/journal.pone.0237315)

PLOS ONE

Testing strategic pluralism: The roles of attractiveness and competitive abilities to understand conditionality in men’s short-term reproductive strategies

--Manuscript Draft--

**Manuscript Number:**	PONE-D-19-27652
**Article Type:**	Research Article
**Full Title:**	Testing strategic pluralism: The roles of attractiveness and competitive abilities to understand conditionality in men’s short-term reproductive strategies
**Short Title:**	The roles of attractiveness and competitive abilities in human reproductive strategies
**Corresponding Author:**	Pablo Polo, Ph.D.<br>Universidad de Playa Ancha<br>Viña del Mar, Valparaíso CHILE
**Keywords:**	human reproductive strategy; Strategic pluralism hypothesis; attractiveness; testosterone
**Abstract:**	The decision to allocate time and energy to find multiple sexual partners or raise children is a fundamental reproductive trade-off. The strategic pluralism hypothesis argues that human reproductive strategies are optionally calibrated towards mating or raising children, according to the expression of features dependent on the individual's condition, such as a selective response to the reproductive trade-off. This study seeks to test predictions derived from this hypothesis in a sample of 243 young men (M ± SD = 22.21 ± 3.20) from Chile’s 5th Region. Specifically, three predictions were considered that raise questions about the relationship between traits related to physical and psychological attractiveness (fluctuating facial asymmetry and self-perception of attractiveness) and competitive skills (baseline testosterone and self-perception of fighting ability) with short-term reproductive strategies. Our results indicate that psychological features related to the self-perception of physical attractiveness and competitive abilities are related to short-term reproductive strategies. It was also established that the effect of fluctuating facial asymmetry on short-term reproductive strategies is fully mediated by the self-perception of physical attractiveness. No evidence was found of any interaction between traits that denote physical attractiveness and those that denote competitive skills. These results support the existing evidence of the importance of facial fluctuating asymmetry as a highly-valued characteristic in terms of sexual selection. They also suggest that traits related to physical attractiveness, in comparison to competitive capabilities, play a relatively more important role in calibrating men’s short-term reproductive strategies.
**Order of Authors:**	Oriana Figueroa
	José Antonio Muñoz-Reyes
	Carlos Rodriguez-Sickert
	Nohelia Valenzuela
	Paula Pavez
	Oriana Ramírez-Herrera
	Miguel Pita
	David Diaz
	Ana Fernández
	Pablo Polo, Ph.D.
**Additional Information:**	
**Question**	**Response**
**Financial Disclosure**	PP was founded by a FONDECYT Iniciación number 11181293 from the Chilean Government. OF was founded by a scholarship for doctoral studies from the Centro de

Powered by Editorial Manager® and ProduXion Manager® from Aries Systems Corporation

Enter a financial disclosure statement that describes the sources of funding for the work included in this submission. Review the submission guidelines for detailed requirements. View published research articles from *PLOS ONE* for specific examples.<br><br>This statement is required for submission and **will appear in the published article** if the submission is accepted. Please make sure it is accurate.<br><br>**Unfunded studies**<br>Enter: *The author(s) received no specific funding for this work.*<br><br>**Funded studies**<br>Enter a statement with the following details:<br>• Initials of the authors who received each award<br>• Grant numbers awarded to each author<br>• The full name of each funder<br>• URL of each funder website<br>• Did the sponsors or funders play any role in the study design, data collection and analysis, decision to publish, or preparation of the manuscript?<br>• **NO** - Include this sentence at the end of your statement: *The funders had no role in study design, data collection and analysis, decision to publish, or preparation of the manuscript.*<br>• **YES** - Specify the role(s) played.<br><br>* typeset	Investigación en Complejidad Social of the Universidad del Desarrollo. The funders had no role in study design, data collection and analysis, decision to publish, or preparation of the manuscript
**Competing Interests**<br><br>Use the instructions below to enter a competing interest statement for this submission. On behalf of all authors, disclose any competing interests that could be perceived to bias this work—acknowledging all financial support and any other relevant financial or non-financial competing interests.<br><br>This statement **will appear in the published article** if the submission is accepted. Please make sure it is accurate. View published research articles from *PLOS ONE* for specific examples.	The authors have declared that no competing interests exist

Powered by Editorial Manager® and ProduXion Manager® from Aries Systems Corporation

**NO authors have competing interests**<br><br>Enter: *The authors have declared that no competing interests exist.*<br><br>**Authors with competing interests**<br><br>Enter competing interest details beginning with this statement:<br><br>*I have read the journal's policy and the authors of this manuscript have the following competing interests: [insert competing interests here]*<br><br>* typeset	
**Ethics Statement**<br><br>Enter an ethics statement for this submission. This statement is required if the study involved:<br><br>• Human participants<br>• Human specimens or tissue<br>• Vertebrate animals or cephalopods<br>• Vertebrate embryos or tissues<br>• Field research<br><br>Write "N/A" if the submission does not require an ethics statement.<br><br>General guidance is provided below. Consult the submission guidelines for detailed instructions. **Make sure that all information entered here is included in the Methods section of the manuscript.**	The research was approved by the ethics committee of Universidad de Playa Ancha. Participants signed a written informed consent prior their participation in the study.

Powered by Editorial Manager® and ProduXion Manager® from Aries Systems Corporation

**Format for specific study types**<br><br>**Human Subject Research (involving human participants and/or tissue)**<br>• Give the name of the institutional review board or ethics committee that approved the study<br>• Include the approval number and/or a statement indicating approval of this research<br>• Indicate the form of consent obtained (written/oral) or the reason that consent was not obtained (e.g. the data were analyzed anonymously)<br><br>**Animal Research (involving vertebrate animals, embryos or tissues)**<br>• Provide the name of the Institutional Animal Care and Use Committee (IACUC) or other relevant ethics board that reviewed the study protocol, and indicate whether they approved this research or granted a formal waiver of ethical approval<br>• Include an approval number if one was obtained<br>• If the study involved *non-human primates*, add *additional details* about animal welfare and steps taken to ameliorate suffering<br>• If anesthesia, euthanasia, or any kind of animal sacrifice is part of the study, include briefly which substances and/or methods were applied<br><br>**Field Research**<br><br>Include the following details if this study involves the collection of plant, animal, or other materials from a natural setting:<br>• Field permit number<br>• Name of the institution or relevant body that granted permission	
**Data Availability**<br><br>Authors are required to make all data underlying the findings described fully available, without restriction, and from the time of publication. PLOS allows rare exceptions to address legal and ethical concerns. See the PLOS Data Policy and FAQ for detailed information.	Yes - all data are fully available without restriction

A Data Availability Statement describing where the data can be found is required at submission. Your answers to this question constitute the Data Availability Statement and **will be published in the article**, if accepted.<br><br>**Important:** Stating 'data available on request from the author' is not sufficient. If your data are only available upon request, select 'No' for the first question and explain your exceptional situation in the text box.<br><br>Do the authors confirm that all data underlying the findings described in their manuscript are fully available without restriction?	
**Describe where the data may be found in full sentences. If you are copying our sample text, replace any instances of XXX with the appropriate details.**<br><br>• If the data are **held or will be held in a public repository**, include URLs, accession numbers or DOIs. If this information will only be available after acceptance, indicate this by ticking the box below. For example: *All XXX files are available from the XXX database (accession number(s) XXX, XXX.*).<br>• If the data are all contained **within the manuscript and/or Supporting Information files**, enter the following: *All relevant data are within the manuscript and its Supporting Information files.*<br>• If neither of these applies but you are able to provide **details of access elsewhere**, with or without limitations, please do so. For example:<br><br>*Data cannot be shared publicly because of [XXX]. Data are available from the XXX Institutional Data Access / Ethics Committee (contact via XXX) for researchers who meet the criteria for access to confidential data.*<br><br>*The data underlying the results presented in the study are available from (include the name of the third party*	All data sets are available from the authors upon request.

and contact information or URL).<br>• This text is appropriate if the data are owned by a third party and authors do not have permission to share the data.<br><br>* typeset	
Additional data availability information:	

Manuscript Click here to download Manuscript Main manuscript.docx

Testing strategic pluralism: The roles of attractiveness and competitive abilities to understand conditionality in men's short-term reproductive strategies

Figueroa[1,2¶], O., Muñoz-Reyes[2¶], J.A., Rodriguez-Sickert[6¶], C., Valenzuela[2,3&], N., Pavez[2&], P., Ramirez[2&], O., Pita[4&], M., Diaz[5&], D., Fernández[4&], A., & Polo[2¶*], P.

[1]Doctorado en Ciencias de la Complejidad Social, Centro de Investigación en Complejidad Social, Facultad de Gobierno, Universidad del Desarrollo, Chile

[2] Laboratorio de Comportamiento Animal y Humano, Centro de Estudios Avanzados, Universidad de Playa Ancha, Chile.

[3]Departamento de Ciencias Biológicas, Facultad de Ciencias Biológicas, Universidad Andrés Bello, Viña del Mar, Chile.

[4]Departamento de Biología, Universidad Autónoma de Madrid, España.

[5]Facultad de Ciencias Económicas, Universidad de Chile.

[6]Centro de Investigación en Complejidad Social, Facultad de Gobierno, Universidad de Desarrollo, Chile.

Corresponding autor: Pablo Polo, Laboratorio de Comportamiento Animal y Humano, Centro de Estudios Avanzados, Universidad de Playa Ancha, Chile. pablo.polo@upla.cl

Contributions: Figueroa O., Polo P. and Muñoz-Reyes J.A. designed the study, interpreted the results and wrote the manuscript. Valenzuela, N., Pavez P., Ramírez O., and Polo P. collected the data. Valenzuela N., Pita M. and Fernández A. analyzed the saliva samples. Díaz D. calculated fluctuating asymmetry. Rodriguez-Sickert translated the manuscript and critically reviewed the first version, and provided a substantial improvement to it. All of the authors also contributed in successive phases to revising the manuscript to improve its technical and scientific quality.

Abstract

The decision to allocate time and energy to find multiple sexual partners or raise children is a fundamental reproductive trade-off. The strategic pluralism hypothesis argues that human reproductive strategies are optionally calibrated towards mating or raising children, according to the expression of features dependent on the individual's condition, such as a selective response to the reproductive trade-off. This study seeks to test predictions derived from this hypothesis in a sample of 243 young men (M ± SD = 22.21 ± 3.20) from Chile's 5th Region. Specifically, three predictions were considered that raise questions about the relationship between traits related to physical and psychological attractiveness (fluctuating facial asymmetry and self-perception of attractiveness) and competitive skills (baseline testosterone and self-perception of fighting ability) with short-term reproductive strategies. Our results indicate that psychological features related to the self-perception of physical attractiveness and competitive abilities are related to short-term reproductive strategies. It was also established that the effect of fluctuating facial asymmetry on short-term reproductive strategies is fully mediated by the self-perception of physical attractiveness. No evidence was found of any interaction between traits that denote physical attractiveness and those that denote competitive skills. These results support the existing evidence of the importance of facial fluctuating asymmetry as a highly-valued characteristic in terms of sexual selection. They also suggest that traits related to physical attractiveness, in comparison to competitive capabilities, play a relatively more important role in calibrating men's short-term reproductive strategies.

Key words: Strategic pluralism, attractiveness, competitive abilities, testosterone, men

Introduction

Reproductive strategies can be defined as an integrated set of adaptations that constitute solutions to different reproductive compromises or trade-offs that the individual faces (1,2). The strategic pluralism hypothesis seeks to explain inter- and intra-individual variation in human reproductive strategies based on the expression of traits dependent on the condition of the individual in interaction with the environment. This hypothesis emphasizes the costs and benefits for men and women with respect to the resources invested in seeking partners versus providing parental care (2,3). The trade-off for men between the search for a partner and parental care is particularly relevant given that men invest less than women in parental care while having a higher potential reproductive rate (4). This means that for men maximizing reproductive success is mainly constrained by the degree of access to multiple reproductive partners (4,5). In this sense, the ability to attract partners and to compete with individuals of the same sex are two factors that affect the resolution of this trade-off, given that they reduce the costs of electing a partner and intrasexual competition, respectively (6). Consequently, the integrated study of these two factors is key to understanding their influence on short-term reproductive strategies.

Physical attractiveness is directly related to the capacity of being chosen as a mate (6–8). Research in this field has identified a series of bodily features associated with attractiveness, fluctuating facial asymmetry being one of these (9,10). Fluctuating facial asymmetry has been proposed as an indicator of genetic quality that reflects the capacity of an individual to maintain a symmetric pattern of stable development (9). It has been observed men with relatively lower levels of fluctuating facial asymmetry are considered more attractive by women, are more economically successful, less faithful and less inclined to invest in their progeny (11–14). These results are consistent with evidence that men with lower levels of fluctuating facial asymmetry have more sexual partners and tend to be more direct in approaching the opposite sex in the context of

courtship, a characteristic that is related to short-term or unrestricted reproductive strategies (2,8,10,15,16). Nevertheless, other investigations (17,18) have not been able to replicate the association between the number of sexual partners and fluctuating facial asymmetry in men. These contradictory findings indicate the need to generate new studies in the field including psychological variables that can influence the relationship between fluctuating facial asymmetry and reproductive success. Particularly relevant for our study is the relationship that has been observed between self-perception of physical attractiveness and the prevalence of short-term reproductive strategies since there is an association between self-perceived characteristics and received social signals, which together affect behavior. That is, the assessment of one's attractiveness is associated with short-term strategy because it reflects the preference of women for certain traits (6,19,20).

At the level of competitive abilities, intrasexual competition is another component that influences access to partners of the opposite sex (6,21). It has been evidenced that testosterone is related to the development of the traits and behaviors related to intrasexual competition, as the display of direct physical aggression (22). T is an androgenizing hormone with two main types of effects: organizational and activational. At the organizational level, it has an androgenizing effect during the prenatal stage and at puberty. At the activational level, baseline T levels, as well as changes in circulating T levels, have been associated with behavioral changes related to intrasexual competition and reproductive efforts (23–25). Focusing on baseline T, there is evidence that baseline T levels are positively related to dominance, aggression, and competitiveness. (23,26). While lower baseline T levels have been associated with men’s relational states and paternity (27,28); that is, baseline T levels are lower in men involved in long-term relationships, and especially in those that are fathers. However, these levels can be expected to remain high in men who, although involved in a relationship, are highly interested in having extramarital relationships (30, 31,32). Puts et al. (2016) studied the relationship between T levels and three dimensions of

sociosexuality: sociosexual desire, sociosexual behavior, and sociosexual attitudes. Their results indicate that T levels are postively related to unrestricted sociosexual psychology (desires and attitudes), which results in a larger numer of reproductive partners (behavior). However, the number of reproductive partners can have a negative effect on T levels by providing a negative feedback mechanism that prevents maintaining high T levels once socio-sexual desires have been satisfied. This suggests that T plays an important role in the willingness of individuals to compete for reproductive partners, primarily through dominance mechanisms, which in turn implies searching for short-term strategies. This relationship is complex because there are negative regulation mechanisms. At the psychological level, testosterone increases competitive behavior and reduces cooperation in determined contexts (26). This suggests that self-perception of fighting ability is positively related to signs of dominance in competitive contexts and that T levels influence intrasexual competition and reproductive success (23,32,33).

Competitive abilities and physical attractiveness do not act independently. Lukaszewski et al. (2014) examined the effect of body strength as an indicator of both fighting ability and attractiveness, and the self-perception of physical attractiveness, as well as the assessment by third parties of physical attractiveness and sociosexual skills and behaviors. Their results show that self-perception of physical attractiveness mediates the positive effect between physical strength, unrestricted socio-sexual attitudes and the number of sexual partners. This indicates the need to explore the way in which indicators of physical attractiveness and fighting ability explain unrestricted human reproductive strategies, using a larger number of morphological, physiological and psychological indicators.

Considering all of the above, we can establish that attractiveness and competitive abilities are important elements of unrestricted reproductive strategies, as well as a possible combined effect of the two when unrestricted reproductive strategies are displayed. However, there have

been few studies that consider biological and psychological (morphological and physiological) variables in an integrated manner in order to understand how reproductive trade-offs are dealt with. The objective of this study is to investigate how the features of physical attractiveness and competitive abilities influence short-term male reproductive strategies. To do this, fluctuating facial asymmetry and the self-perception of physical attractiveness are considered as anthropometric and psychological features of physical attractiveness. Likewise, the levels of circulating baseline testosterone and self-perception of fighting ability are considered physiological and psychological features associated with competitive capacity. It can thus be expected that i) there is a positive relationship between physical attractiveness and short-term reproductive strategies, and a negative relationship between fluctuating facial asymmetry and the self-perception of attractiveness. The psychological variable (self-perception of attractiveness) is expected to mediate the effect of the physical variable (fluctuating facial asymmetry) on short-term strategies, ii) that there is a positive relationship between baseline T, self-perception of fighting ability and short-term reproductive strategies. Finally, when analyzed as a whole, iii) the individual's competitive abilities are expected to moderate the effect of attractiveness on short-term reproductive strategies. In this way, attractiveness has a positive effect on short-term reproductive strategies that is greater for individuals who are highly competitive.

Materials and methods

Participants: The initial sample was composed of 246 young men. However, three individuals were rejected because they failed to complete the questionnaires, so the sample was reduced to 243 men between 18 and 36 years of age (*M* ± *DE* = 22.21 ± 3.20). The participants were recruited using ads posted in universities in the 5th Region of Chile. In terms of sexual orientation, 97.5% stated they were heterosexual, 3.5% stated they were homosexual, and 53.8% stated they were in a couple at the time of participating in the investigation.

The research was approved by the ethics committee of the sponsoring university. The participants received an economic incentive of a payment of five thousand Chilean pesos (approximately seven US dollars) as an economic incentive for participating, plus up to 30,000 pesos (approximately 43 USD and almost twice of the daily minimum wage) conditional on their performance in the economic games that the study comprised.

Psychological measurements

Sociosexual orientation scale (SOI): We used a multidimensional version of the SOI developed by Jackson & Kirkpatrick (2007) that had been applied previously with Chilean subjects (see Polo, et al., 2019). The scale is divided into attitudinal and behavioral dimensions. There are two attitudinal factors that measure sociosexual orientation in the short-term (e.g. *"I can easily imagine being comfortable with and enjoying casual sex with different women"*, 10 items) and long-term (e.g. *"I am interested in maintaining a long-term romantic relationship with a special woman"*, 7 items). These dimensions are in the format of 7-point Likert scale responses in which 1 indicates "strongly disagree" and 7 indicates "strongly agree". The behavioral dimensions consisted of 5 items of open-ended responses that included questions about the number of sexual partners in the past (3 items) (e.g. *"Over your entire life, how many women have you had complete sexual relations with?*), a question about sexual fantasies *(How often do you fantasize about having sexual relations with women other than your current partner?)* and a question about the expected number of sexual partners in the future *(How many women do you think you will have sexual relations with in the next five years*). This study only considered the items referring to the attitudinal factor in short-term relationships. Polo et al. (2019) obtained a Cronbach α value of .95 for the aforementioned factor, while a Cronbach α of .70 was obtained in the present study, indicating that the instrument is sufficiently reliable.

Self-perceived fighting ability questionnaire (SPFAQ): We used a version of the SPFAQ developed by Muñoz-Reyes et al. (2012), which had been applied previously with Chilean subjects (see Muñoz-Reyes, Fernández, Flores-Prado, Guerra, & Turiégano, 2015). This is a short 4-question questionnaire that assesses the self-perception of fighting skills (1. How good a fighter am I? 2. How do others perceive my abilities as a fighter? 3. How much fear can I provoke in someone who is about to fight me? 4. What are my odds of winning a fight if I have to fight someone?). The responses are on a seven-point Likert scale in which 1 indicates "well below average" and 7 indicates "well above average." Muñoz-Reyes, Fernández, Flores-Prado, Guerra, & Turiégano (2015) obtained a Cronbach α score of .84 in the original study. In the present study, an α coefficient of .87 was obtained, indicating adequate reliability for the studied sample.

Self-perception of attractiveness: This consists of a single question to assess self-perception of physical attractiveness (How attractive do you think you are?). The response is on a 7-point Likert scale in which 1 indicates "not attractive at all" and 7 indicates "very attractive".

Anthropometric measurements

Fluctuating facial asymmetry: This indicator of attractiveness is measured according to the protocol of Sanchez-Pages & Turiegano (2010). Frontal photographs were taken of the participants with a Nikon D-90 camera. Participants were asked not to wear any form of facial adornment and to maintain as neutral an expression as possible. Photos where the subject smiled or inclined his head were rejected. Fluctuating facial asymmetry was calculate based on 106 facial points or landmarks (LM), which were obtained with the program FACE ++ (see Kosinski, 2017 for a similar procedure; Zhou, Fan, Cao, Jiang, & Yin, 2013). This software identifies high-precision facial reference points, like facial contours, eyes, eyebrows, and nose. The use of this software was automated with a MatLab software algorithm connected to the interface of programming applications of FACE ++. Fluctuating facial asymmetry was determined with the software MorphoJ (Klingenberg, 2011; also

see http://www.flywings.org.uk/MorphoJ_page.htm) based on the Procrustes distances between each LM the corresponding mirror image LM. These distances were then broken down by Procrustes analysis into directional asymmetry and fluctuating asymmetry (39,40).

Baseline testosterone: Baseline testosterone was measured with a 1-ml sample of saliva of the participants, who were asked not to eat or drink anything other than water for at least one hour before the sample was taken. The samples were taken at approximately noon to avoid alterations in testosterone levels as the result of circadian hormonal variation. A passive saliva collection method (Salimetrics®) was used to collect samples. After being collected, the samples were centrifuged, frozen and stored at -20°C in cyrotubes (SalivaBio®) for 20 days. All the samples were analyzed with the Testosterone Enzyme Immunoassay Kit (Salimetrics®) in accordance with manufacturer's instructions. Due to a problem with storing the saliva samples (freezing rupture and consequent increase in temperature), 136 individuals were not included in the baseline testosterone analysis because the intra and interplate variation coefficients were very high. Thus, the sample for all the baseline testosterone analyses was 107 individuals. The coefficients of intraplate and interplate variation were respectively equal to and less than 15%.

Statistical analysis: A general linear model was applied in a first stage for the first hypothesis. Self-perception of attractiveness and fluctuating facial asymmetry were considered independent variables and short-term socio-sexual orientation was considered the dependent variable. The age and relational status of the subjects were also considered as control variables. The analysis of the residues showed that the variables are not distributed normally in the model. We transformed the variable of short-term sociosexual orientation to its quadratic form in order to tackle the issue of normality. To the extent that the results with and without transformed variables did not vary in terms of their statistical significance, we only report the results without the transformation. A mediation analysis was applied in the second stage, in which fluctuating facial asymmetry was

considered as an independent variable and short-term socio-sexual orientation as a dependent variable, and self-perception of attractiveness was considered a mediating variable. The age and relationship status of subjects were also considered.

For the second hypothesis, a general linear model was applied in which self-perception of fighting abilities and baseline testosterone levels were considered independent variables. As with hypothesis 1, the age and relational status of the subjects were included as control variables. Because of the reduced sample size with the introduction of the baseline T variable and because it did not have a statistically significant effect on the model, the variable was eliminated to restore the complete sample. Even though the residues in the latter model were normally distributed, an extreme outlier was identified that was confirmed with the statistical Tukey test, because of which the subject was eliminated. The value of the outlier was -37.10, which is lower than the limit considered by the Tukey test (-37,10 < -8.3543 – 1.5*19.058; -8.3543 being the first quartile and 19.058 the interquartile range). Finally, for the third hypothesis, a general linear model tested whether there was an interaction between fighting abilities and the self-perception of physical attractiveness, controlling again for age and the relational status of the subjects on short-term reproductive strategies. The normality of residues was verified for the second and third hypothesis. IBM SPSS 21.0 software was used for the general linear models and PROCESS software was used for the mediation analyses (41) . The level of significance was set at $\alpha = .05$.

Results

Table 1 shows the descriptive statistics for the morphological, physiological and psychological variables. A general linear model as shown in Table 2 was applied for the first hypothesis. The results indicate a positive effect of the self-perception of attractiveness (B= 2.42, p= .007) on unrestricted reproductive strategies (measured using the short-term sociosexual

orientation scale), as shown in Figure 1. However, FFA did not appear to have any effect on unrestricted reproductive strategies (*B*= 198.91, *p*= .291).

Table 1: Descriptive statistics (M±DE) for the entire sample for the variables, SPFAQ, SPA, short-term SOI, FFA.

**Variable**	**N**	**M ± DE**
SPFAQ	243	16.82 ± 4.88
SPA	243	4.68 ± .96
Short-term SOI	243	44.30 ± 13.47
FFA	243	.01 ± .00

Note: Self-perceived fighting ability questionnaire (SPFAQ), self-perception of attractiveness (SPA), Short-term sociosexual orientation Instrument (Short-term SOI). Fluctuating facial asymmetry (FFA)

Table 2: General linear model for short-term SOI, n=243 considering partner, age, SPA and FFA as variables

**Parameter**	**B**	**Typical error**	**T**	**Sig.**	η_p^2
Intersection	21.20	7.54	2.81	.005	.032
Partner= 1	-3.53	1.68	-2.09	.037	.018
Age	.47	.27	1.72	.087	.012
SPA	2.42	.89	2.72	.007	.030
FFA	198.91	187.84	1.05	.291	.005

Note: Partner=1 (with a partner n=130), self-perception of attractiveness (SPA), fluctuating facial asymmetry (FFA)

Figure 1. Dispersion diagram representing the relationship between self-perceived physical attractiveness and the short-term SOI score in function of individual relational status. The scores represent the observed values for individual without partners (empty circles) and with partners (filled circles). The lines represent the values estimated by the model in the range for individuals without partners (dashed line) and with partners (solid line). Age and fluctuating facial asymmetry were assessed in their mean values.

An analysis was then made with fluctuating facial asymmetry, self-perception of attractiveness, and short-term SOI. Figure 2 shows the observed results of measurement by the model. There was an indirect effect of FFA on short-term reproductive strategies (*B*= -69.18, *BootSE*= 44.65), with a confidence interval of 95%, calculated using a bootstrapping procedure, with a lower limit of -173.47 and an upper limit of -2.75. There is no significant direct effect of FFA on short-term SOI (*B*= 198.92, *p*= .291). The effect of FFA is fully captured by self-perception of attractiveness.

Figure 2. Unstandardized regression coefficients between FFA and short-term SOI mediated self-perception of attractiveness controlling for age and relational status, n=243. The standard errors are shown in parentheses.

Note: Fluctuating facial asymmetry (FFA), self-perception of attractiveness (SPA), short-term socio-sexual orientation instrument (short-term SOI) *p<.05; **p< .01; ***p< .001

Table 3 shows the results for the second prediction, an effect of competitive abilities was found (β= .62, *p*= .016), but no effect of baseline T was found as an explanatory variable of short-term reproductive strategies (β= .01, *p*= .510). These observations were made for the reduced sample (n=107).

Table 3: General linear model 1 for short-term SOI, n=107 considering partner, age, SPFAQ, and baseline T as variables

**Parameter**	**B**	**Typical error**	**T**	**Sig.**	η_p^2
Intersection	25.95	11.97	2.16	.033	.044
PARTNER=1	-5.59	2.79	-2.00	.048	.038
AGE	.33	.42	.79	.429	.006
SPFAQ	.62	.27	2.44	.016	.055
Baseline T	.012	.01	.66	.51	.004

Note: Partner=1 (with a partner), Age, Self-perceived fighting ability questionnaire (SPFAQ) and baseline testosterone (baseline T).

Given that no effect of baseline T was established, a second model was tested for the complete sample (n=242) excluding this variable. In this case, a significant effect of self-perceived competitive abilities was found (*SPFAQ*; *B*= .38, *p*= .030, see Table 4 and Figure 3)

Table 4: General linear model 2 for short-term SOI, n= 242, considering partner, age, and SPFAQ as variables, and eliminating the outlier of the residues from the sample

**Parameter**	**B**	**Typical error**	**T**	**Sig.**	η_p^2
Intersection	27.77	6.38	4.35	.000	.073
PARTNER=1	-4.44	1.68	-2.63	,009	.028
AGE	.58	.26	2.21	.028	.020
SPFAQ	.38	.17	2.18	.030	.020

Note: Partner=1 (with a partner), Self-perceived fighting ability questionnaire (SPFAQ).

Figure 3. Dispersion diagram depicting the relationship between the self-perception of fighting capacity and the short-term SOI score in function of the relational status of the individuals. The score represents the observed values for individuals without a partner (empty circles) and with a partner (filled circles). The lines represent the values estimated by the model in the range observed for individuals without partners (dashed line) and with partners (solid line). The age was estimated at the mean value.

The effects of the variables in the model were tested for the third prediction, and a positive effect was found for self-perceived attractiveness (B = 1.89, p = .041) on short-term strategies, as shown in Table 5. We tested to determine if there is an interaction between attractiveness traits (based on self-perceived attractiveness) and fighting abilities (based on the SPFAQ questionnaire). As shown in Table 6, no effect was observed for the interaction between the two variables on short-term strategies.

Table 5: General linear model 2 for short-term SOI, n= 242, considering partners, age, SPFAQ and SPAQ as variables

**Parameter**	**B**	**Typical error**	**T**	**Sig.**	η_p^2
Intersection	23.19	6.97	3.32	.001	.045
PARTNER=1	-4.37	1.68	-2.59	.010	.028
AGE	.47	.28	1.73	.084	.013
SPFAQ	.25	.18	1.40	.160	.008
SPA	1.89	.92	2.05	.041	.018

Note: Partner=1 (with a partner). Self-perceived fighting ability questionnaire (SPFAQ). Self-perception of attractiveness (SPA).

Table 6: General linear mode 3 for short-term SOI, n= 242, considering partners, age, SPFAQ and SPA, and the interaction between SPA and SPFAQ as variables

**Parameter**	**B**	**Typical error**	**t**	**Sig.**	η_p^2
Intersection	46.04	15.18	3.03	.003	.038
Partner=1	-4.53	1.68	-2.69	.008	.030
AGE	.48	.27	1.76	.078	.013
SPFAQ	-1.16	.85	-1.35	.177	.008
SPA	-2.95	3.00	-.98	.327	.004
SPA * SPFAQ	.29	.17	1.69	.092	.012

Note: Partner=1 (with a partner). Self-perceived fighting ability questionnaire (SPFAQ). Self-perception of attractiveness (SPA).

Discussion

The strategic pluralism hypothesis explains the conditionality of human reproductive strategies and the resolution of the trade-off between investment in multiple partners and investment in parental care (42). This hypothesis considers that there are biological, psychological and anthropometric factors that calibrate reproductive behavior according to the context in which the individual faces the aforementioned trade-off. This study proposes three partially supported hypotheses. Specifically, it emphasizes the role of competitive abilities and physical attractiveness in men on the unfolding of unrestricted reproductive strategies (short-term strategies at the scale

of sociosexual orientation). The main result indicates that the traits of attractiveness have a greater effect on unrestricted reproductive strategies than does fighting abilities.

The first hypothesis sought to determine if there is a positive relationship between physical attractiveness, as measured by fluctuating facial asymmetry and the self-perception of attractiveness, and unrestricted reproductive strategies. Our results suggest that fluctuating facial asymmetry has no effect, while self-perceived attractiveness does have an effect. Simpson et al., (1999) found similar results for fluctuating facial asymmetry, and were unable to establish a relationship between this variable and unrestricted sociosexuality. In contrast to Simpson et al. (1999), our investigation involved conducting mediation analysis, which allowed us to establish for the first time that the relationship between fluctuating facial asymmetry and unrestricted reproductive strategies can be fully mediated by the self-perception of attractiveness. This result is consistent with those of other investigations that have found the effects of morphological features on the psychology of unrestricted male sociosexual behavior (for example, facial masculinization in Kruger, 2006; corporal strength in Lukaszewski et al., (2014); and skeletal muscle and strength in Kordsmeyer, Hunt, Puts, Ostner, & Penke, 2018). Therefore, more physically attractive men in this study, that is, men with lower fluctuating facial asymmetry, displayed unrestricted reproductive strategies, which in turn was mediated by the self-perception of attractiveness. This supports the theoretical assumptions of the strategic pluralism theory (2) by maintaining that attractive men opt for maximizing short-term reproductive partners over increasing parental investment. This makes senses, especially if we consider that fluctuating facial asymmetry has been associated with the implementation of short-term reproductive strategies (16), with the number of sexual partners over one’s lifetime (45,46), and with the perception of attractiveness (10). All of the above makes evident the role of physical attractiveness, and in particular of fluctuating facial asymmetry, in reproductive success. it has hypothesized that this importance is due to its evolutionary value, representing a

proxy for good genetic quality (2,6), which could be a signal to females for a male's inclination towards short-term relationships.

For the second hypothesis, we expected to find an effect of the markers of competitive abilities, measured using the self-perception of competitive abilities (SPFAQ) and baseline testosterone, on short-term reproductive strategies. An effect on unrestricted strategies was found for self-perception of fighting ability, but not for baseline T. Other studies have established a relationship between fighting and mate value (35), which is defined as "the complete set of characteristics that an individual has in a given moment and in a particular context that affects his capacity to successfully find, attract and keep a partner" (47). According to Muñoz-Reyes et al., (2015) , fighting ability is associated with the mate value of a partner, which implies a positive relationship between this variable and men's assessments of their chances of finding partners, and therefore of employing intrasexual competition strategies, which implies a high degree of self-confidence in the search for partners. It has been established that the self-perception of fighting ability is also associated with aggressive behavior (22). These findings, together with the results of our investigation, indicate that it is plausible to support that fighting ability is a conflict resolution mechanism in situations of intrasexual competition, which is consistent with studies that have found a positive association between traits associated with fighting abilities and reproductive success ((20,35,48). No effect was found for baseline testosterone on short-term reproductive studies, as had been expected. Studies have associated testosterone with the search for social status (49), self-confidence in competitive situation (33) and the adoption of dominant roles in economic environments (26). Consequently, testosterone can be considered a social hormone associated with status-seeking and not so much with aggression in itself. Status in turn could be related to different reproductive strategies according to the way it is acquired. The relationship between testosterone and reproductive strategies has been explored in other studies and evidence has been found that

favors the role of testosterone as a promoter of short-term strategies. For example, Edelstein, Chopik, & Kean, (2011) found an interaction between unrestricted sociosexuality and the relational state of men, and established that men in relationships with partners, but that have interest in extramarital relationships, have similar testosterone levels as those of single men, producing a positive attitude about unrestricted strategies. Puts et al. (2016) established that there is a negative relationship between of sexual partners and baseline T, and a positive relationship between high levels of baseline T and unrestricted sociosexual psychology (desire and attitudes). Although this investigation employed a reduced version of the sociosexual orientation questionnaire (50), a relationship was found between baseline T levels and an orientation toward short-term strategies. The reduced sample in the model that assesses the effect of baseline T on reproductive strategies could explain the null result with respect to this variable.

On the basis of the third hypothesis, a relationship was expected between attractiveness (measured by the self-perception of attractiveness) and competitive abilities (measured by the self-perception of fighting abilities) on unrestricted strategies. A significant effect of self-perception of attractiveness was found, although not of fighting skills, which supports the results of previous studies (e.g., Muñoz-Reyes et al., 2015) that fighting skills are an important feature at the time of engaging in intrasexual competition, but there are variables linked to attractiveness (such as self-perception) that have a greater effect in terms of intersexual selection for the case of unrestricted strategies (6). In this sense, competitive abilities can be understood as a set of features that make up attractiveness and that together exercise an effect on men's unrestricted reproductive strategies.

Among the limitations of this research is the inclusion of only one anthropometric measurement (fluctuating facial asymmetry), which, although a robust measurement to study physical attractiveness, could be complemented with others that are also considered attractive

features. Another limitation was the loss of data due to the storage of samples and handling of the T kit, despite following protocols tested in other investigations. Other anthropometric variables associated with unrestricted strategies should be included in future research, such as facial masculinization (30), height (e.g. Polo, Fernandez, Muñoz-Reyes, Dufey, & Buunk, 2018) and body mass (e.g. Polo et al., 2019).

In conclusion, the present study contributes evidence to the hypothesis of strategic pluralism with respect to the importance of features of attractiveness to explain unrestricted reproductive strategies among men and where competitive skills are configured as a relevant mechanism in acting out intrasexual competition strategies. These findings support the hypothesis of strategic pluralism, which explains the reproductive trade-off that men have between maximizing the number of sexual partners and investing in parental care, considering the importance of the interaction among anthropometric features on the self-perception (that is, psychological features) of subjects when the reproductive trade-off is solved.

Acknowledgements

This project was possible thanks to FONDECYT Iniciación grant No. 11181293 from an agency of the Chilean Government. We are also grateful for a scholarship for doctoral studies from the Centro de Investigación en Complejidad Social of the Universidad del Desarrollo, which made the work of Oriana Figueroa in this project possible.

References

1. Polo P, Muñoz-Reyes JA, Pita M, Shackelford TK, Fink B. Testosterone-dependent facial and body traits predict men's sociosexual attitudes and behaviors. Am J Hum Biol [Internet]. 2019;(October 2018):e23235. Available from: https://onlinelibrary.wiley.com/doi/abs/10.1002/ajhb.23235
2. Gangestad SW, Simpson JA. The evolution of human mating: trade-off and strategic pluralism. Vol. 23, Behavioral and Brain Sciences. 2000.
3. Schacht R, Borgerhoff Mulder M. Sex ratio effects on reproductive strategies in humans. R Soc Open Sci [Internet]. 2015;2(1):140402–140402. Available from: http://rsos.royalsocietypublishing.org/cgi/doi/10.1098/rsos.140402
4. Trivers RL. Parental investment and sexual selection. In: Sexual Selection and the Descent of Man: The Darwinian Pivot. 1972. p. 136–79.
5. Clutton-Brock, T., & Parker GA. The Quarterly Review of Biology. Rev Lit Arts Am. 1992;67(4):437–56.
6. Buss DM, Schmitt DP. Mate Preferences and Their Behavioural Manifestations. 2018;(September 2018):1–34. Available from: http://www.springerlink.com/content/gxkv6265668t4t12/%5Cnpapers2://publication/uuid/BFAFEEFA-9EED-4EAE-AF7A-E6859A2BA8C3
7. Dion K, Berscheid E, Walster E. What is beautiful is good and more accurately understood. Physical attractiveness and accuracy in first impressions of personality. Psychol Sci [Internet]. 1972;24(3):285–90. Available from: http://www.ncbi.nlm.nih.gov/pubmed/21051521
8. Little AC, Jones BC, Debruine LM. Facial attractiveness: Evolutionary based research. Philos Trans R Soc B Biol Sci. 2011;366(1571):1638–59.

9. Muñoz-Reyes JA, Pita M, Arjona M, Sanchez-Pages S, Turiegano E. Who is the fairest of them all? The independent effect of attractive features and self-perceived attractiveness on cooperation among women. Evol Hum Behav [Internet]. 2014;35(2):118–25. Available from: http://dx.doi.org/10.1016/j.evolhumbehav.2013.11.005

10. Van Dongen S, Gangestad SW. Human fluctuating asymmetry in relation to health and quality: A meta-analysis. Evol Hum Behav [Internet]. 2011;32(6):380–98. Available from: http://dx.doi.org/10.1016/j.evolhumbehav.2011.03.002

11. Hernández-López L, García-Granados DM, Chavira-Ramírez R, Mondragón-Ceballos R. Testosterone, the progesterone/estradiol ratio, and female ratings of masculine facial fluctuating asymmetry for a long-term relationship. Physiol Behav [Internet]. 2017;175:66–71. Available from: http://dx.doi.org/10.1016/j.physbeh.2017.03.033

12. Shackelford TK, Schmitt DP, Buss DM. Universal dimensions of human mate preferences. Pers Individ Dif. 2005;39(2):447–58.

13. Stephan P. How Economics Shapes Science. 2015;(March):384. Available from: http://www.hup.harvard.edu/catalog.php?isbn=9780674088160

14. Scheib JE, Gangestad SW, Thornhill R. Symmetry and Cues of Good Genes. Proc R Soc London B Biol Sci. 1999;266(1431):1913–7.

15. Simpson JA, Gangestad SW. Individual differences in sociosexuality: Evidence for convergent and discriminant validity. J Pers Soc Psychol [Internet]. 1991;60(6):870–83. Available from: http://doi.apa.org/getdoi.cfm?doi=10.1037/0022-3514.60.6.870

16. Simpson JA, Christensen PN, Leck K, Gangestad SW. Fluctuating asymmetry, sociosexuality, and intrasexual competitive tactics. J Pers Soc Psychol. 1999;76(1):159–72.

17. Kordsmeyer TL, Penke L. PT. 2017; Available from: http://dx.doi.org/10.1016/j.evolhumbehav.2017.08.002

18. Rhodes G. The Evolutionary Psychology of Facial Beauty. Annu Rev Psychol. 2006;57:199–226.

19. Back MD, Penke L, Schmukle SC, Asendorpf JB. Knowing your own mate value: Sex-specific personality effects on the accuracy of expected mate choices. Psychol Sci. 2011;22(8):984–9.

20. Lukaszewski AW, Larson CM, Gildersleeve KA, Roney JR, Haselton MG. Condition-dependent calibration of men's uncommitted mating orientation: Evidence from multiple samples. Evol Hum Behav. 2014;35(4):319–26.

21. Buss y Schmit, 1993.pdf.

22. Muñoz-Reyes JA, Gil-Burmann C, Fink B, Turiegano E. Physical strength, fighting ability, and aggressiveness in adolescents. Am J Hum Biol. 2012;24(5):611–7.

23. Geniole SN, Bird BM, Ruddick EL, Carré JM. Effects of competition outcome on testosterone concentrations in humans: An updated meta-analysis. Horm Behav [Internet]. 2017;92:37–50. Available from: http://dx.doi.org/10.1016/j.yhbeh.2016.10.002

24. Carré JM, Archer J. Testosterone and human behavior: the role of individual and contextual variables. Curr Opin Psychol. 2018;19:149–53.

25. Archer J. Testosterone and human aggression: An evaluation of the challenge hypothesis. Neurosci Biobehav Rev. 2006;30(3):319–45.

26. Mehta PH, Lawless DesJardins NM, van Vugt M, Josephs RA. Hormonal underpinnings of status conflict: Testosterone and cortisol are related to decisions and satisfaction in the hawk-dove game. Horm Behav [Internet]. 2017;92:141–54. Available from: http://dx.doi.org/10.1016/j.yhbeh.2017.03.009

27. Gray PB& BCC. Human male testosterone, pair-bonding and fatherhood. In: Endocrinology. 2012. p. 270–93.

28. Edelstein RS, Chopik WJ, Kean EL. Sociosexuality moderates the association between testosterone and relationship status in men and women. Horm Behav [Internet]. 2011;60(3):248–55. Available from: http://dx.doi.org/10.1016/j.yhbeh.2011.05.007

29. Puts, Pope, Hill, Cárdenas, Welling, Wheatley and B. Fulfilling Desire: Evidence for negative feedback between men's testosterone, sociosexual psychology, and sexual partner number. Horm Behav. 2015;70:14–21.

30. Gangestad SW, Thornhill R, Garver-Apgar CE. Men's facial masculinity predicts changes in their female partners' sexual interests across the ovulatory cycle, whereas men's intelligence does not. Evol Hum Behav [Internet]. 2010;31(6):412–24. Available from: http://dx.doi.org/10.1016/j.evolhumbehav.2010.06.001

31. Pollet T V., der Meij L van, Cobey KD, Buunk AP. Testosterone levels and their associations with lifetime number of opposite sex partners and remarriage in a large sample of American elderly men and women. Horm Behav [Internet]. 2011;60(1):72–7. Available from: http://dx.doi.org/10.1016/j.yhbeh.2011.03.005

32. Mehta PH, van Son V, Welker KM, Prasad S, Sanfey AG, Smidts A, et al. Exogenous testosterone in women enhances and inhibits competitive decision-making depending on victory-defeat experience and trait dominance. Psychoneuroendocrinology [Internet]. 2015;60:224–36. Available from: http://dx.doi.org/10.1016/j.psyneuen.2015.07.004

33. Eisenegger C, Kumsta R, Naef M, Gromoll J, Heinrichs M. Testosterone and androgen receptor gene polymorphism are associated with confidence and competitiveness in men. Horm Behav [Internet]. 2017;92:93–102. Available from: http://dx.doi.org/10.1016/j.yhbeh.2016.09.011

34. Jackson JJ, Kirkpatrick LA. The structure and measurement of human mating strategies: toward a multidimensional model of sociosexuality. Evol Hum Behav. 2007;28(6):382–91.

35. Muñoz-Reyes JA, Fernández AM, Flores-Prado L, Guerra R, Turiégano E. Fighting ability influences mate value in late adolescent men. Pers Individ Dif. 2015;80:46–50.

36. Sanchez-Pages S, Turiegano E. Testosterone, facial symmetry and cooperation in the prisoners' dilemma. Physiol Behav [Internet]. 2010;99(3):355–61. Available from: http://dx.doi.org/10.1016/j.physbeh.2009.11.013

37. Zhou E, Fan H, Cao Z, Jiang Y, Yin Q. Extensive facial landmark localization with coarse-to-fine convolutional network cascade. Proc IEEE Int Conf Comput Vis. 2013;386–91.

38. Kosinski M. Facial Width-to-Height Ratio Does Not Predict Self-Reported Behavioral Tendencies. Psychol Sci. 2017;28(11):1675–82.

39. Klingenberg CP, Barluenga M, Meyer A. Asymmetry SHAPE ANALYSIS OF SYMMETRIC STRUCTURES : QUANTIFYING VARIATION AMONG INDIVIDUALS AND ASYMMETRY. Society [Internet]. 2002;56(10):1909–20. Available from: http://www.bioone.org/perlserv/?request=get-abstract&doi=10.1554%2F0014-3820(2002)056%5B1909%3ASAOSSQ%5D2.0.CO%3B2

40. Klingenberg CP, McIntyre GS. Geometric Morphometrics of Developmental Instability: Analyzing Patterns of Fluctuating Asymmetry with Procrustes Methods. Evolution (N Y). 1998;52(5):1363.

41. Hayes AF. Introduction to Mediation, Moderation, and Conditional Process Analysis: A Regression-based Approach . 2013. 507 p.

42. Gangestad SW, Simpson JA. The evolution of human mating : Trade-offs and strategic pluralism. 2000;573–644.

43. Kruger DJ. Male facial masculinity influences attributions of personality and reproductive strategy. Pers Relatsh. 2006;13(4):451–63.

44. Kordsmeyer TL, Hunt J, Puts DA, Ostner J, Penke L. The relative importance of intra- and

intersexual selection on human male sexually dimorphic traits. Evol Hum Behav [Internet]. 2018;39(4):424–36. Available from: https://doi.org/10.1016/j.evolhumbehav.2018.03.008

45. Gangestad SW, Thornhill R. The evolutionary psychology of extrapair sex: The role of fluctuating asymmetry. Evol Hum Behav. 1997;18(2):69–88.

46. Thornhill R, Gangestad SW. Human fluctuating asymmetry and sexual behavior. Psychol Sci. 1994;5(5):297–302.

47. Fisher M, Cox A, Bennett S, Gavric D. Components of self-perceived mate value. J Soc Evol Cult Psychol. 2014;2(4):156–68.

48. Archer J, Thanzami V. The relation between physical aggression, size and strength, among a sample of young Indian men. Pers Individ Dif. 2007;43(3):627–33.

49. Eisenegger C, Haushofer J, Fehr E. The role of testosterone in social interaction. Trends Cogn Sci [Internet]. 2011;15(6):263–71. Available from: http://dx.doi.org/10.1016/j.tics.2011.04.008

50. Penke L, Asendorpf JB. Beyond Global Sociosexual Orientations: A More Differentiated Look at Sociosexuality and Its Effects on Courtship and Romantic Relationships. J Pers Soc Psychol. 2008;95(5):1113–35.

51. Polo P, Fernandez A, Muñoz-Reyes JA, Dufey M, Buunk AP. Intrasexual Competition and Height in Adolescents and Adults. Evol Psychol. 2018;16(1):1–8.

Figure1

Click here to download Figure Figure1.tif

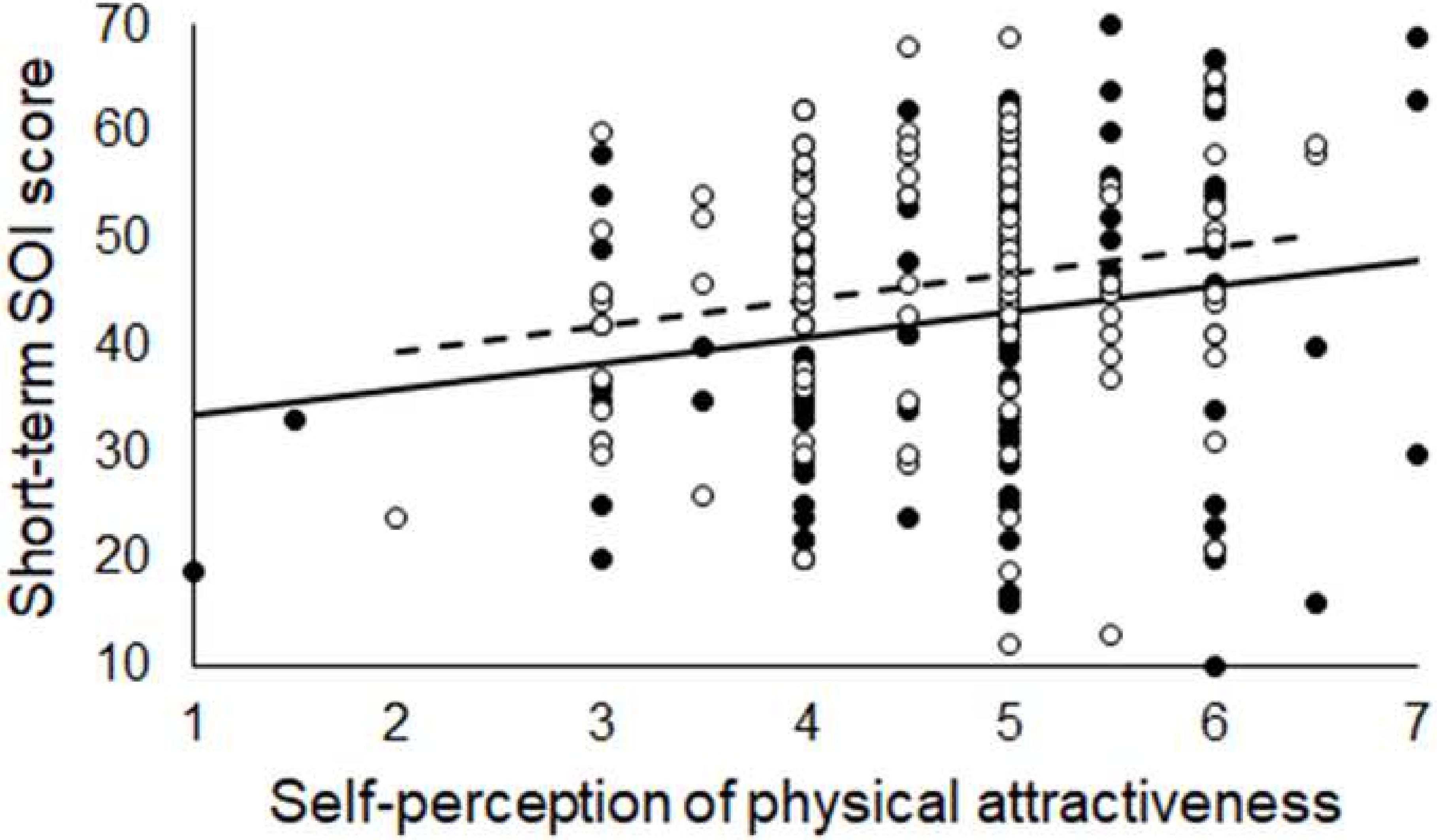

 Click here to download Figure Figure2.tif

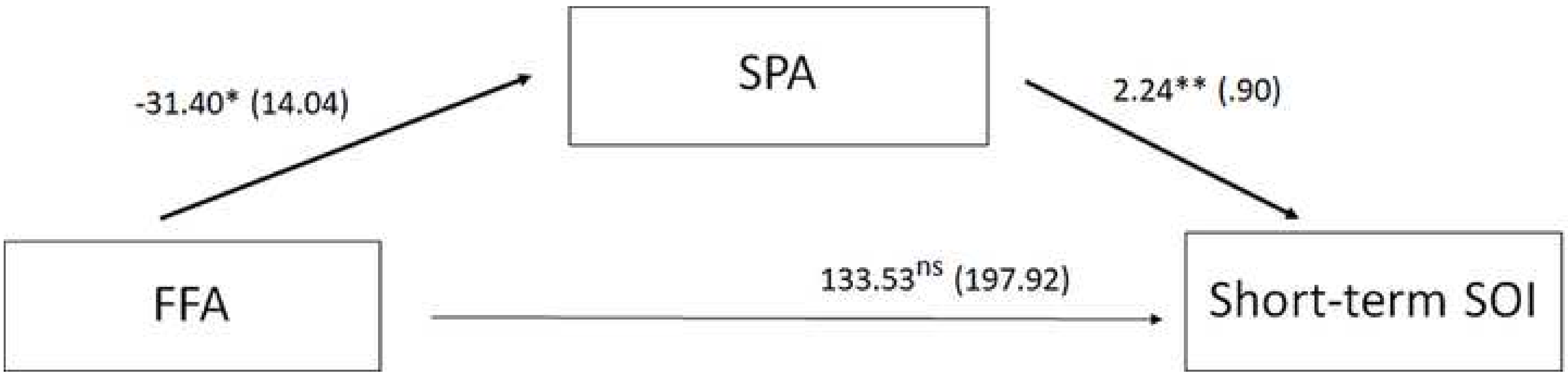

 Click here to download Figure Figure3.tif

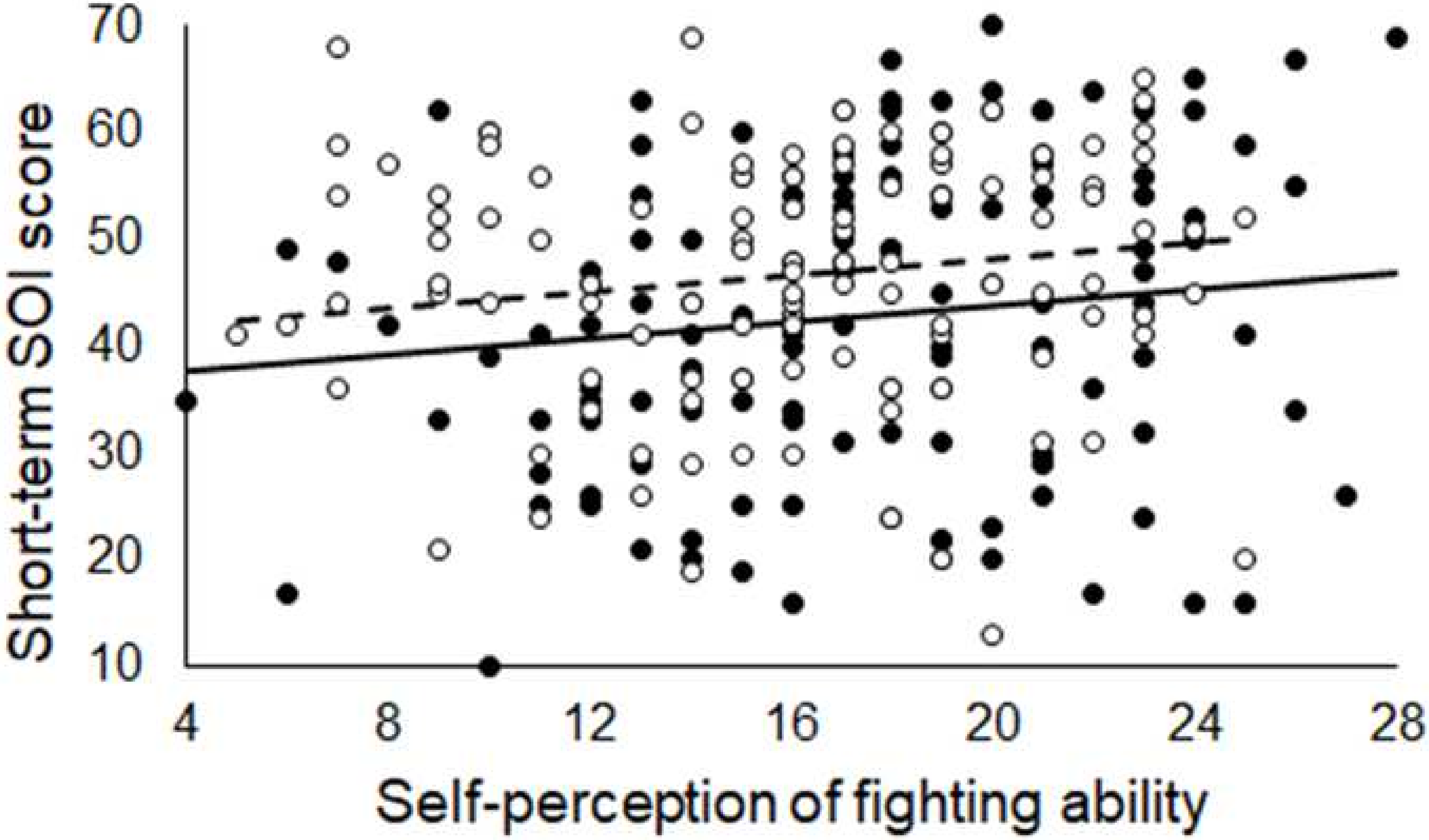

Supplement: Attachment — Submitted filename: PONE-D-19-27652_reviewer comments.pdf [file pone.0237315.s004.pdf]
