# Supplementary material for: Testing strategic pluralism: The roles of attractiveness and competitive abilities to understand conditionality in men’s short-term reproductive strategies
Source: PLoS One. 2020 Aug 31;15(8):e0237315. doi: 10.1371/journal.pone.0237315 (PMC7458284; doi:10.1371/journal.pone.0237315)
Supplement: Attachment — Submitted filename: PONE-D-19-27652_R1 review.pdf [file pone.0237315.s006.pdf]

PONE-D-19-27652_R1

Testing strategic pluralism: The roles of attractiveness and competitive abilities to understand conditionality in men's short-term reproductive strategies

Oriana Figueroa, José Antonio Muñoz-Reyes, Carlos Rodriguez-Sickert, Nohelia Valenzuela, Paula Pavez, Oriana Ramírez-Herrera, Miguel Pita, David Diaz, Ana Fernández, Pablo Polo

The manuscript is much improved over the original.

I have to respectfully disagree with this statement: "However, fluctuating asymmetry is the population-level measure of developmental instability, that accounts with the more robust theoretical support as a measure quality. In this sense, all individuals in population exhibit a determined level of fluctuating asymmetry, being the more symmetrical individuals, which possess the values near to zero. Therefore, the higher fitness males possess the lower values in fluctuating asymmetry. Directional asymmetry and antisymmetry usually have been considered as the result of strong genetic effect (e.g., 1)."

Imagine two populations: high-fitness individuals (blue) and low-fitness individuals (orange). The two curves differ in their variances. But in both cases, the modal individuals are still perfectly symmetrical. There are just fewer perfectly symmetrical individuals among the low-fitness population. Now imagine a transition from FA (blue curve) to antisymmetry (bimodal distribution) in the low-fitness individuals. If the low-quality individuals have an antisymmetric distribution of individual asymmetries, then most individuals are asymmetrical. There is abundant evidence for such transitions, even in the very first paper on fluctuating asymmetry by Kenneth Mather. When Mather selected for increased asymmetry in Drosophila, the population transitioned from FA to antisymmetry. And the heritability of antisymmetry and directional asymmetry is only slightly greater than that of fluctuating asymmetry, so the statement that such asymmetries are "the result of strong genetic effect" doesn't hold up to scrutiny. See the very detailed papers by Larry Leamy. In some of my own (unpublished) research I've been able to document a transition from FA to antisymmetry by knocking down (RNAi) the activity of a single gene. These were in inbred lines with little genetic variation.

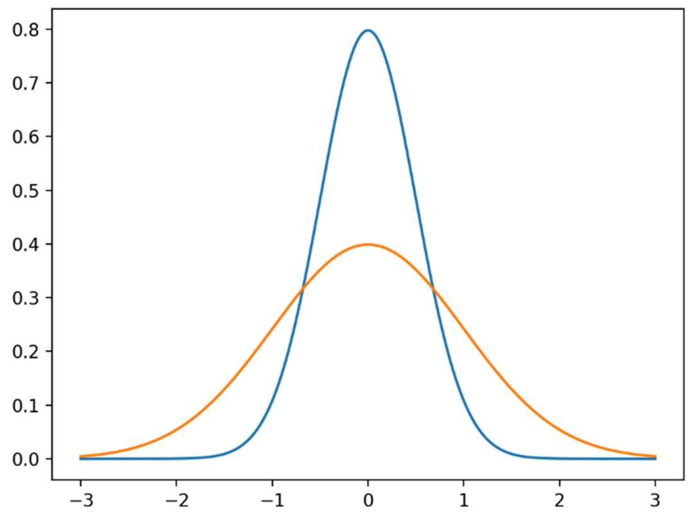

Regardless, the authors need to document that the statistical distributions they are dealing with are symmetrical, unimodal, and with a mean of zero. Does a normal distribution fit the data? One just cannot wish away these other forms of asymmetry, and they may be important.
