# Supplementary material for: Testing strategic pluralism: The roles of attractiveness and competitive abilities to understand conditionality in men’s short-term reproductive strategies
Source: PLoS One. 2020 Aug 31;15(8):e0237315. doi: 10.1371/journal.pone.0237315 (PMC7458284; doi:10.1371/journal.pone.0237315)
Supplement: Attachment — Submitted filename: Response to Reviewers.docx [file pone.0237315.s005.docx]

RESPONSE TO EDITOR AND REVIEWERS COMMENTS

PONE-D-19-27652
Testing strategic pluralism: The roles of attractiveness and competitive abilities to understand conditionality in men’s short-term reproductive strategies
PLOS ONE

Dear Dr Polo,

Thank you for submitting your work to PLOS ONE, and apologies for the delay in returning the manuscript to you. I have now secured the opinion of two reviewers in the field. As you will see from their comments, they generally find much to like in the manuscript, commenting on the large sample size, the number of theoretically driven measures, and the testing of an influential theory in the area. They also highlight different concerns about the manuscript, which I agree with. The concerns of Reviewer 1 centred around the presentation of the hypotheses of the SPH, which was presented as an essentially dichotomous outcome for a given male, when in reality it is likely to be more continuous. The bearing on the results and interpretation should be considered. Reviewer 1 also raised concerns over the measurement, definition, and accuracy of the use of facial asymmetry as a proxy for attractiveness perception. This is something I also agree with, and have researched myself (e.g. Jones and Jaeger, 2019; Symmetry). I do not think that these are insurmountable issues and careful discussion and interpretation of the measures is required.

R: We totally agree about the continuous nature of human reproductive strategies. We change some parts in the introduction be clearer about this In addition, we also have addressed the issues about fluctuating asymmetry as a reliable signal of quality and attractiveness. More details about the changes performed can be found in the answers given to reviewer 1.

Reviewer 2 pointed out some more statistical issues with the manuscript, as well as the use of economic games which was puzzlingly mentioned once, and then is not touched upon again. I agree with Reviewer 2 in that much more detail is needed about the background of the work here. In my opinion, and after reading the manuscript, perhaps the most problematic part of the manuscript were the analyses and the modelling procedure. Reviewer 2 also pointed this out and I am in full agreement with them on this point; that there are too many models here testing too many hypotheses.

R: We have provided more details about the background and answered the reviewer 2 concerns about the procedure.

The main issue is that while you have sufficient data and variables to test a model of short-term SOI approaches, splitting variables across into different models obviously ignores the fact that in reality, these variables were measured from, and exist within, the sample of males you obtained. The data-generating process (DGP) is not being accurately tested here. In my view, you have the capacity to run build two models - one which includes all predictors and your full sample, which includes any theoretically important interactions that you wish to test, and another which is includes testosterone as a measure on the reduced sample for which those measures are available for. This way, you can be more certain in your conclusions about what predictors affect what in the presence of other variables, rather than splitting things up. Alternatively, you may wish to first account for the variation explained by age and partner status, and fit your models in a stepwise fashion. Either way, I do not think the current analysis is convincing in its conclusions as it fails to approximate the DGP. I also wondered about the transformations used here. Squaring a variable if it is somewhat non-normal will not make it more normal, but will lead to a kind of gamma-distributed variable (most values bunched up to one side of the distribution), but in any case the mathematics of ordinary least squares does not mind non-normally distributed variables for the predictors or the response; only that the residuals are normally distributed. This is mentioned implicitly but I couldn't tell if the residuals were normal or something was wrong with the predictors.

I was unsure about the use of the mediation analysis here. There are significant reservations about the use of mediation analysis in cross sectional designs - for mediation to be conclusive multiple time points are required, or at least with a cross sectional design heavily theoretical arguments and multiple measures used as mediators should be used to build confidence in the results. I think a far safer conclusion could be made by specifying an interaction between FA and SPA - for example, do men with lower levels of FA perceive themselves to be particularly attractive, and thus have a specific SOI score? To test this would require a simple interaction and centring of your variables.

R: Thanks for your comments. We have followed your advice and have changed our analyses to avoid mediation. Our original proposal was that more symmetric individuals were expected to show higher short-term sociosexual orientation partially because they perceived themselves as highly attractive. It is reasonable to think that it exists a causal relationship between FA and self-perception of physical attractiveness, although it is true that the later can be affected by multiple factors in addition to FA. A similar reasoning was made for testosterone and self-perception of fighting abilities. In addition, previous studies (e.g. Lukaszewsky et al., 2014), proposed a mediation effects between variables of attractiveness and fighting ability on short-term sociosexual orientation. However, as you mention, mediation analysis with cross-sectional data produces estimators that are biased and this is problematic, especially with full mediation effects. Consequently, we decided to change our proposal and consider now that psychological variables are expected to moderate the effect of FA and testosterone. This decision has produced major changes in the manuscript, but we think that it has gained in methodological rigor.

1.	Lukaszewski AW, Larson CM, Gildersleeve KA, Roney JR, Haselton MG. Condition-dependent calibration of men’s uncommitted mating orientation: evidence from multiple samples. Evol Hum Behav. 2014;35(4):319-26. doi: 10.1016/j.evolhumbehav.2014.03.002

The manuscript was well written and has a high quality data set (which I would encourage the authors share via the OSF, if they can), and I look forward to receiving a resubmission that addresses these concerns.

**5. Review Comments to the Author**


Reviewer #1: This study gathers together evidence in support of the Strategic Pluralism Hypothesis, the idea that males can allocate time and resources to either mate selection or the raising of children. The study is well done and believable, but the paper can be improved especially in the presentation of the Strategic Pluralism Hypothesis and its predictions.

The Strategic Pluralism Hypothesis is presented first, followed by a set of the sub-hypotheses. These sub-hypotheses are actually predictions of the Strategic Pluralism Hypothesis, but is unclear how they are related. They also need to be portrayed as predictions. For example, if SPH is true, then we expect a, b, and c to be true. Finally, what are the alternatives to the SPH? Science functions best when there are alternative explanations for phenomena.

R: We have clarified and justified the predictions proposed this study based on expectations from the SPH. In addition, based on a suggestion from the editor, we have modified the analytical approach since mediation analysis applied to cross-sectional data may be problematic. The predictions now are based on moderation effects rather than mediation effects. We agree that an alternative hypothesis would enrich the paper, but we did not include them in the paper for several reasons. First, from an evolutionary perspective, the Strategic Pluralism Hypothesis and the Sexual Strategies Theory are the two main theoretical frameworks that aim to explain the variability in human reproductive strategies. However, both proposals are not incompatible rather they focus in different aspects of human reproductive strategies. We selected the SPH because is focused more in within sex variation and flexibility in the behavioral manifestations and we were interested in these aspects in this paper. Second, the other theoretical framework that aim to explain the variability in human reproductive strategies is the Social Role Theory. Unfortunately, our research design was not developed to test the influence of cultural of social norms in the expression of these behaviors as to do so we had needed a sample with a significant variation in cultural background and in the gender roles. Moreover, our study is aimed in within sex variability, and Social Role Theory mainly focuses in between sex differences.

The Strategic Pluralism Hypothesis is posed as an either/or affair, when it is really an entire spectrum. The two ends of the spectrum are 100% of the time and energy spent trying to mate with every available partner versus 100% of the time spent raising children with one partner. But the time and energy allocations might be 50/50 or 70/30. If the optimal allocations for males are 50/50, how would that effect the predictions? Would one be able to detect an effect? And although it isn’t mentioned in the text, sexually transmitted diseases might enter into the balance of selective forces as well.

R: We totally agree that reproductive tactics are a spectrum. Indeed, we decided to employ the SOI questionnaire developed by Jackson & Kirkpatrick because, in our knowledge, it is the only one that considers the attitudes towards unrestricted (short-term) and restricted (long-term) sociosexuality as two independent factors. In this paper, we focused on short-term reproductive tactics and postulated that the traits considered are affecting the predisposition of individuals to invest in short-term partners. Accordingly, we expected a higher unrestricted sociosexuality for those individuals with higher expression of attractive and competitive traits, regardless of their restricted sociosexuality. We have rewritten some parts in the introduction to make clear this point and to add ecological and social features as additional selective forces that play a role in the mentioned trade-off.

Facial symmetry is employed as an indicator of facial attractiveness. The authors portray this symmetry/asymmetry as fluctuating asymmetry. But fluctuating asymmetry is a population parameter. Individual asymmetry may collectively represent fluctuating asymmetry, or it may represent directional asymmetry or antisymmetry or a mixture of all three kinds of asymmetry. The authors mention directional asymmetry when they mention the morphometric analysis and Procrustes distance, but that is the last time it appears. Was there detectable directional asymmetry?

R: We employed Proclustes ANOVA analysis to decomposed variance in shape due to directional and fluctuating asymmetry. Therefore, directional asymmetry was detectable and took into account when calculating individual values of fluctuating asymmetry through this methodology. We have added more information about how we calculated individual values of fluctuating asymmetry in methods.

The other two forms of asymmetry (directional asymmetry and antisymmetry) may be better indicators of male quality than fluctuating asymmetry. If low fitness males exhibit fluctuating asymmetry, the modal low fitness males will still be perfectly symmetrical. But if low fitness is associated with a transition from fluctuating asymmetry to directional asymmetry (or antisymmetry), then the predictive value of individual asymmetry is much better. There is a literature for this and evidence for transitions among the three forms of asymmetry in populations.

R: We agree with referee that there are three kinds of population asymmetries: fluctuating asymmetry, directional asymmetry, and antisymmetry. However, fluctuating asymmetry is the population-level measure of developmental instability, that accounts with the more robust theoretical support as a measure quality. In this sense, all individuals in population exhibit a determined level of fluctuating asymmetry, being the more symmetrical individuals, which possess the values near to zero. Therefore, the higher fitness males possess the lower values in fluctuating asymmetry. Directional asymmetry and antisymmetry usually have been considered as the result of strong genetic effect (e.g., 1). A better example for this is handedness in human being and their effect over biomechanical development of arm muscularity. As we stated before, we have followed the typical procedure of split fluctuating asymmetry form directional asymmetry, we knew that there is new proposal to integrate different kind of symmetry (2), however, we prefer to use the more validated methodology.

On the other hand, we agree with referee that there is evidence from transitions between fluctuating asymmetry to directional asymmetry in human being, for example for the orbital opening (3), but these analyses are centered in the study of symmetry from the effect of environmental or biological variables. We have not included these predictions in our theoretical model, because we are studying the effect over a determined behavior, and not the causes of fluctuating asymmetry. Therefore, we have not estimated transitions in symmetry, and have used the typical estimation.

Finally, despite that we consider fluctuating asymmetry as a measure of developmental instability, we mention in the new version of the manuscript that there is controversy about the importance of fluctuating asymmetry as a marker of this instability.

1. Özener, B. (2010). Fluctuating and directional asymmetry in young human males: effect of heavy working condition and socioeconomic status. *American journal of physical anthropology*, *143*(1), 112-120.
2. Ekrami, O., Claes, P., White, J. D., Weinberg, S. M., Marazita, M. L., Walsh, S., ... & Dongen, S. V. (2020). A multivariate approach to determine the dimensionality of human facial asymmetry. *Symmetry*, *12*(3), 348.
3. Tomaszewska, A., Kwiatkowska, B., & Jankauskas, R. (2015). Is the area of the orbital opening in humans related to climate?. *American Journal of Human Biology*, *27*(6), 845-850.

Throughout the text, the authors mention that fluctuating facial asymmetry is associated with attractiveness. This is a misleading way to portray this association, because it is symmetry, not asymmetry, that is considered attractive. Moreover, it is “individual” symmetry (or asymmetry) that potential mates respond to. Fluctuating asymmetry is assumed to underlie the variation in individual asymmetries. This is an assumption that must be tested, but it rarely is. At the very least it needs to be mentioned.

R: Thanks for the comment. In the reviewed manuscript, we explicitly mention that we assume that fluctuating asymmetry is underlying individual differences in asymmetry. In addition, we clarify that it is facial symmetry rather than asymmetry the feature that is considered attractive.

In addition, the evidence that fluctuating asymmetry is an indicator of genetic quality has been long discussed, and without resolution. It would pay to study (and cite) some of the papers by population geneticists. They generally aren’t sympathetic to the idea of “genetic” quality. How would you measure it? In what sense might a male have “quality” genes? This brings us to Darwinian and inclusive fitness. There are very few studies that have actually looked at fluctuating asymmetry and all possible fitness components (ability to find a mate, fertility, etc.).

R: In the new version of the manuscript, we acknowledge the controversial issue about the consideration of fluctuating asymmetry as an indicator of genetic quality. In addition, we have replaced the term genetic quality with a more general term of individual quality.

For the measurement of fluctuating asymmetry, what is the measurement error associated with the approach? All studies of FA need to assess measurement error, because it can inflate estimates of FA. To do this, the authors need to take more than one photo of each person and then use FACE ++ and MorphoJ to estimate overall asymmetry and follow that with a variance component ANOVA. The methodology in this section is extremely unclear. I presume that a single, unitless number is produced (Procrustes distance), with larger numbers indicating more asymmetry.

R: Following to our ethical protocol, we selected one picture of each individual and calculation of fluctuating asymmetry were performed on them. In former studies, two researchers placed the landmarks in each individual (39 landmarks) and we calculated error from this source. This time we decided to employ FACE++ to increase the number of landmarks to 106, but as the points were placed always in the same place for each picture, we lack measurement error.

The methodology has been clarified. Fluctuating asymmetry was calculated as the deviation of each individual’s asymmetry from the overall average asymmetry. To do this first, we calculated Proclustes distances that reflect both directional and fluctuating asymmetry. Then, we employed a Proclustes ANOVA to decompose variance in these two types of asymmetries. Accordingly, we obtained a single measure of fluctuating asymmetry for each individual with larger numbers indicating more fluctuating asymmetry.

For the statistical analysis, the relationship between the Strategic Pluralism Hypothesis and its predictions is unclear. You need to make clear, for example, that the SPH predicts a relationship between socio-sexual orientation and perception of attractiveness and facial symmetry. Moreover, there are no alternative hypotheses mentioned in the paper. If the results didn’t support SPH, what would they support?

R: We have clarified and justified the predictions proposed this study based on expectations from the SPH. We agree that alternative hypothesis would enrich the paper, but we did not include them in the paper for several reasons. First, from an evolutionary perspective, the Strategic Pluralism Hypothesis and the Sexual Strategies Theory are the two main theoretical frameworks that aim to explain the variability in human reproductive strategies. However, both proposals are not incompatible; rather, the focus is on different aspects of human reproductive strategies. We selected the SPH because it is focused more in within sex variation and flexibility in the behavioral manifestations, and we were just interested in this aspect in this paper. Second, the other theoretical framework that aims to explain the variability in human reproductive strategies is the Social Role Theory. Unfortunately, our research design was not developed to test the influence of cultural of social norms in the expression of these behaviors as to do so we had needed a sample with a significant variation in cultural background and in the gender roles. Moreover, our study is aimed in within sex variability, and Social Role Theory mainly focuses in between sex differences.

The English is grammatically good, though somewhat wordy. In several places, I found the meaning of a phrase or sentence to be obscure. This is usually attributable to wordiness and passive voice.

R: Thanks for the comment. We have tried to avoid passive voice and wordiness in this new version.

In the figures, it would be more effective to spell out SOI, FFA, and SPA. I was finding myself having to look these up every time I came across them.

R: In this new version, we avoided the use of acronyms.

I have also included many comments on the manuscript itself.

R: Thanks, we followed almost all your comments and suggestions. The exception is L77 in which we mention that men with lower levels of fluctuating asymmetry tend to be more direct in approaching the opposite sex. We agree with your comment, but we do not mention the proximal causes of this behavior so we decided to keep the sentence unchanged.

Reviewer #2: The authors have conducted a novel test of a well-established theory that adds to knowledge in this area, contains different measures (e.g., biological, psychometric) and is conducted on a decent sized sample that may be deemed ‘non-WEIRD’. The manuscript is of interest to scholars in different fields. The authors find relationships between self-perceived attractiveness, self-perceived fighting ability and openness toward short-term sexual relationships, albeit one that may differ according to the model used (e.g. attractiveness appears to be more stable predictor).

Based on the information reported, I deem there no ‘fatal flaws’ in the work, but would suggest the work requires ‘major revision’ in light of the points below.

General method
I was a little bit concerned that this study appears to be part of an experiment involving economic games - this clearly functions as a competitive task but does not seem to be anywhere in the manuscript (unless I’ve missed something). Personally it seems like something that should be part of the current paper (regardless of whether it constitutes a ‘failed manipulation’) as it could feasibly alter testosterone levels (particularly with the size of the potential reward offered)? A good justification would be required for why this is treated as a ‘separate study’ and/or explanation of how it was part of the general study session. On a second point, you control for relationship status in your analyses but have a sample of men who have responded to items such as ‘….other than your current partner’ when around half the sample are single. I’m not sure if this represents an issue to consider/explain (see next point).

R: The general procedure involved economic games that were aimed to measure cooperative and aggressive tendencies as a part of a wider project. These games were played after the participants answered the questionnaires. Consequently, answers in the questionnaires were unaffected by games. Basal levels of testosterone were measured before participants played the games. But, as you mention, we unknow whether the knowledge of the potential reward, that was mentioned in the ads, affected testosterone levels. We have added more information about the games in the section of method and discuss the potential effect of the reward in levels of testosterone as a limitation of our study.

Regarding the second point, that question is the only one that considers paired individuals in its original formulation. We employed the same question but during the explanation we indicated that individuals that were not paired could answer that question considering how often they fantasize about having sex with women or men (according to their sexual orientations).

Statistical models and their relation to hypotheses
The way you setup your argument, I’m expecting to see a single model to test the relative/unique contribution of ‘mate quality’ and ‘resource holding potential’ on short term sexual strategies. You sort-of do this (and take into account the different sample sizes when including testosterone in the model). But at no point do you include symmetry in the same model as the predictors related to competitive ability, and I’m not really sure why. The use of the term ‘competitive’ (when describing perceived fighting ability as a proxy for competitiveness, like a personality trait) is a bit odd as people who consider themselves fighters might not be ‘competitive’ per se (i.e., they might not have to try as hard because they are good fighters). Finally on this issue, given the SOI questionnaire (see above) and effects of your control variable (relationship status), I did wonder why the predicted effects were not explored to see if they interacted with partnership status (i.e., stronger among single men, who are more likely to be competing for mates).

R: We did not include fluctuating asymmetry in the model including competitive ability because we found that its effect was fully mediated by self-perception of physical attractiveness. In any case, following a suggestion from the editor, we have modified the analytical approach since mediation analysis applied to cross-sectional data may be problematic, especially with regards to full mediation. Then, we investigated moderation effects rather than mediation effects in this new version. In addition, we have reduced the models to two, one considering a reduced data set and including measures of basal testosterone, and another with the full data set and excluding measures of testosterone. Each model was fitted in three successive steps in order to investigate main effects and the predicted interactions.

We agree with your comment about that good fighters may be not show a competitive personality, but our point is that being a good fighter reduces costs involved in intrasexual competition because may be associated to greater chances to win a potential fight or to signals that intimidate rivals. We have tried to clarify this in the text.

Finally, we initially expected that traits related to attractiveness and competitive abilities would be positively associated to short-term sociosexual orientation in single and paired individuals, although global levels of short-term sociosexual orientation could be affected by relationship status. The reason is that individuals in a relationship may be actually in an uncommitted relationship and then, competing and looking for mates. Nevertheless, we explored the interactions between partnership status and the predicted effects and were not significant.

Stylistically, I found the analyses hard to follow, which was a problem as there are multiple models, and some effects altered beyond conventional significance in different models/with different control variables. I would suggest reporting the analytical strategy as each model is reported in the results. Then, depending on your response to my earlier point, I would suggest omitting tables and reporting all stats within-text, moving the stats from Table 1 to the appropriate point in the methods (or at the very least, provide a single table with sub-headings to show how the model is ‘built’).

R: We have reduced the models and we hope that analyses are easier to follow now.

Discussion
Based on above, I would revisit this section for clarity, so that the conclusions match the models, particularly your point on line 307 and line 330 (i.e., you don’t measure reproductive success – only one subscale of the SOI is applicable here and even this could be deemed ‘reproductive potential’ rather than ‘reproductive success’). ‘Three partially supported hypotheses’ is a bit vague/subjective in light of my reading of your results thus far. Line 348 is an important oversight (i.e., why did you examine testosterone if you did not expect it to be related to ‘reproductive strategies’). You then say on line 363 that there is an effect of testosterone! I would also relate the concluding paragraph more directly to the study data.

R: Due to the analytical changes, discussion has suffered a large amount of modifications. We agree, the sentence in line 348 is confusing, we meant to say that we did not find any effect of testosterone conversely of we had expected. We deleted the last part of the sentence to avoid confusions.

Minor points.
• Second sentence of abstract (better wording?)

R: We have tried to improve clarity.

• Line 46 – ‘highly valued’ implies a strong relationship between asymmetry and attractiveness (it exists across studies, but some papers find the relationship tends to be small in effect size).

R: This sentence has been changed.

• Around line 96 – I would suggest brief discussion of some null findings for balance, e.g. work by Michal Kandrik. I would suggest briefly discussing Quist et al. 2012 in the Discussion (as she finds that high SOI women prefer male facial symmetry).

R: Thanks for the comment. We have included null findings and stressed that the effect depends on the context.

• Line 100 – ‘more interested’ rather than ‘highly interested’ (the latter may imply a strong effect).

R:Thanks, we replaced the word.

• Page 5, first paragraph – I was struggling to follow the logic of the argument here – why many sexual partners would reduce testosterone (and any accompanying citation), why you are arguing that these men are only competing for short-term partners, and why citation # 26 is then followed by discussion of self-perceived fighting ability. Please could you unpack/clarify?

R: We have modified this paragraph in order to be clearer in our argument.

• Just as a general thought - it’s a shame self-rated attractiveness wasn’t anchored to an ‘average’ in the same way that the fighting scale was.

R: In this new version, we have centered all the predictor variables to their means.

• In the tables, I haven’t heard of the phrase ‘typical error’ – do you just mean ‘standard error’?

R: Sorry, it was a mistake. We meant to indicate standard error.

• First paragraph of discussion – a bit ‘wordy’ (please clarify/unpack).

R: We tried to make clear this paragraph.

General proofreading
Lines 33 (optionally); 36 (fifth region – plus specify roughly where this is – also applies elsewhere with ‘5th’); 66 (electing); 62 (please refer to males rather than men if Trivers is the citation); use of terms ‘relational’ and ‘residue’ (versus ‘relationship status’ and ‘residuals’); 291 (‘tested to determine’); 326 (senses).

R: Thank you for the proofreading

Thanks for the opportunity to review this interesting research.
