# Supplementary material for: Testing strategic pluralism: The roles of attractiveness and competitive abilities to understand conditionality in men’s short-term reproductive strategies
Source: PLoS One. 2020 Aug 31;15(8):e0237315. doi: 10.1371/journal.pone.0237315 (PMC7458284; doi:10.1371/journal.pone.0237315)
Supplement: Attachment — Submitted filename: Response to reviewers.docx [file pone.0237315.s007.docx]

RESPONSE TO REVIEWERS

PONE-D-19-27652R1
Testing strategic pluralism: The roles of attractiveness and competitive abilities to understand conditionality in men’s short-term reproductive strategies

6. Review Comments to the Author

Please use the space provided to explain your answers to the questions above. You may also include additional comments for the author, including concerns about dual publication, research ethics, or publication ethics. (Please upload your review as an attachment if it exceeds 20,000 characters)

Reviewer #1: The manuscript is much improved over the original.

I have to respectfully disagree with this statement: “However, fluctuating asymmetry is the population-level measure of developmental instability, that accounts with the more robust theoretical support as a measure quality. In this sense, all individuals in population exhibit a determined level of fluctuating asymmetry, being the more symmetrical individuals, which possess the values near to zero. Therefore, the higher fitness males possess the lower values in fluctuating asymmetry. Directional asymmetry and antisymmetry usually have been considered as the result of strong genetic effect (e.g., 1).”

Imagine two populations: high-fitness individuals (blue) and low-fitness individuals (orange). The two curves differ in their variances. But in both cases, the modal individuals are still perfectly symmetrical. There are just fewer perfectly symmetrical individuals among the low-fitness population. Now imagine a transition from FA (blue curve) to antisymmetry (bimodal distribution) in the low-fitness individuals. If the low-quality individuals have an antisymmetric distribution of individual asymmetries, then most individuals are asymmetrical. There is abundant evidence for such transitions, even in the very first paper on fluctuating asymmetry by Kenneth Mather. When Mather selected for increased asymmetry in Drosophila, the population transitioned from FA to antisymmetry. And the heritability of antisymmetry and directional asymmetry is only slightly greater than that of fluctuating asymmetry, so the statement that such asymmetries are “the result of strong genetic effect” doesn’t hold up to scrutiny. See the very detailed papers by Larry Leamy. In some of my own (unpublished) research I’ve been able to document a transition from FA to antisymmetry by knocking down (RNAi) the activity of a single gene. These were in inbred lines with little genetic variation.

[See the figure in the attached file.]

Regardless, the authors need to document that the statistical distributions they are dealing with are symmetrical, unimodal, and with a mean of zero. Does a normal distribution fit the data? One just cannot wish away these other forms of asymmetry, and they may be important.

R: Thanks for your comment. We have analyzed the distribution of differences between the original and the reflected landmarks from each individual from the coordinates in the asymmetry component provided by the MorphoJ after the Procrustes fit. For the x’s coordinates, we found that 44 out of 48 distributions were normally distributed and for the y’s coordinates, 45 out of 48 distributions were normally distributed. We mentioned these distributions in the methods section. Most of the distributions were not around 0 (40 out 48 for the x-axis and 35 out 48 for the y-axis). This result is in accordance with the significant effect of “side” in the Procrustes ANOVA analysis denoting the existence of directional asymmetry; however, our measure of fluctuating asymmetry took into account the directional asymmetry as we describe in the method section. Normality tests, descriptives and t-tests are reported in the attached file. We appreciate your critical feedback and now the manuscript reflects more accurately the controversy about the significance of fluctuating asymmetry as a reliable indicator of quality. In addition, it is relevant to establish that we have not found articles were antisymmetry was linked to behaviour in human beings. However, there is evidence that supports the presence of a certain degree of antisymmetry from skulls; therefore, we agree with the decision to include these analyses in the manuscript.

Reviewer #2: I am happy that the authors have addressed my concerns, except for the following relatively minor points:

• For completeness, please could you include brief critical details on the photography procedure, term the payment as ‘reimbursement’ rather than ‘incentive’, and provide (in-text) the age characteristics of the smaller sample who had testosterone measured.

R: Thanks for your suggestions. We have added information about the focal length, speed, aperture and light conditions in which the pictures were taken. We have changed the word incentive and provide more information about age in the reduced data set.

• The manuscript reads well. One more proofread would be beneficial, e.g., for typos (e.g., ‘physical’ misspelt) wording that is ‘hyperbolic’ (e.g. describing something as having a ‘great’ influence when the effect may be small/moderate), and three overly-complex passages:

R: A general proofread has been made.

“according to the expression of features dependent on the individual's condition, such as a selective response to the reproductive trade-off”

R: We have deleted the last part of the sentence. Our intention was to signal that facultative calibration of reproductive strategies can be understood as an adaptative response to the trade-off, but it is not critical information and deleting it reduced the complexity of the sentence.

“but there are variables linked to attractiveness (such as self-perception) that have a greater effect in terms of intersexual selection for the case of unrestricted strategies”

R: We have amended the last part of this paragraph since some of the argumentation was related to former results.

I also couldn’t understand the final concluding paragraph (except for its first sentence) – which also refers to mediation, which you no longer do.

R: I am confused about your comment here. First, because in the first sentence of the last paragraph, we do not mention mediation. We mention mediation in the last sentence of the paragraph (maybe you meant to say last instead of first?), but here our point is to signal that designs that are more suitable to study mediation effects may contribute to understand the role of fluctuating asymmetry in future studies. Anyway, we have tried to be clearer in this paragraph.

Hypothesis 1 also seems oddly worded: “fluctuating asymmetry should be negative associated with short-term reproductive strategies, especially in individuals with high levels of self-perceived physical attractiveness…” – by that I mean it would read better if it referred to ‘facial symmetry’ – as I think you’re trying to get across here that this POSITIVE relationship (with symmetry) would be stronger in individuals who think of themselves as attractive (as they are better able to offset any costs of engaging in short-term mating competition)?

R: We agree that it could be less confusing the use of symmetry instead of fluctuating asymmetry, however, we prefer to keep the term fluctuating asymmetry in the predictions because there are two more sources of asymmetry (directional and antisymmetry) and consequently, fluctuating asymmetry and facial symmetry are not 100% equivalent. In the introduction, we mention facial symmetry when talking about the relationship between symmetry and attractiveness, which includes fluctuating asymmetry.

• You explained in the response that single individuals could answer one of the items differently on the SOI, which seems OK. I don’t think you report this in the manuscript, though. Related to this (and my earlier point on partnership status), please carefully do a final proofing check on analyses in light of the major changes made to the manuscript. For example, you’ve said in the response document that there were no effects of relationship status, but there does appear to be an effect in Table 3.

R: Regarding the question about sociosexual desire “How often do you fantasize about having sex with someone other than your current dating partner?” I agree with you that seems to be intended to paired individuals (indeed I was imprecise in my first response), but the main aim of this item is to assess sexual attraction that is specifically targeted at potential mates to whom no committed romantic relationship exists. Since many single participants asked us that if they had to answer the question, we included in the general explanation a particular clarification on this point. We did not mention that because we only considered the short-term factor of the sociosexual attitude dimension due to the aim of this study.

Regarding relationship status, sorry if my previous response was not clear, but we meant to indicate that we explore potential interactions between relationship status and predictor variables (fluctuating asymmetry, self-perceived psychical attractiveness, basal levels of testosterone and self-perceived fighting ability) and we did not find any interaction effect. The result in Table 3 is related to the main effect of relationship status. In any case, we have carefully checked our analysis, and no inconsistencies in the results were found.

• I leave this as the Editor’s decision, but personally I thought the in-text results could be even more concise – as you seem to be reporting everything in tables, perhaps the text could simply refer to what was/was not significant, cross-reference to the tables, and save page space by not reporting sets of statistical values twice. Please could you also format (italicize) all statistical values.

R: Thanks, we have amended the format of statistical values.

• Apologies if I’ve missed this, but do you refer anywhere to the scale end points for facial fluctuating asymmetry (and what high/low scores mean), just to get a general sense when reading it of how variable the sample were (variability seems quite low according to the descriptive statistics).

R: Facial fluctuating asymmetry was measured as the deviation of each individual’s asymmetry from the overall average asymmetry. This deviation represents a distance (unitless) in Euclidean space (Procrustes distance), therefore the minimum value is 0 (no deviation from the overall average asymmetry) and the value increases with the deviation from the overall average asymmetry. We have clarified the scale for facial fluctuating asymmetry in the manuscript.

• It might be useful to mention around limitations, briefly, that any null effects of basal testosterone in the current study don’t necessarily rule out relationships between T changes and sexual/competitive behaviours (e.g. if elicited experimentally via a confederate or other manipulation). Please also very briefly mention, in light of my prior comments, that the later competitive tasks might represent a small limitation introducing noise into the current study (i.e., they know they are attending at some point to engage in a competitive task and are being reimbursed a reasonable amount to do so, albeit these tasks are after the measures taken for the current study).

R: We have included the suggested limitation in our discussion.

We appreciate all your critical comments and suggestions.
